# Complex Degradation Mechanisms Accessible to Anion Exchange Membrane Ionomers on Model Catalysts, NiO and IrO_2_

**DOI:** 10.1021/acselectrochem.5c00040

**Published:** 2025-04-29

**Authors:** Mai-Anh Ha, Emily K. Volk, Oliver Leitner, Avital Isakov, Héctor J. González Vélez, Shaun Alia, Ross Larsen

**Affiliations:** † Computational Science Center, 53405 National Renewable Energy Laboratory, 15013 Denver West Parkway, Golden, Colorado 80401, United States; ‡ Chemistry and Nanoscience Center, 53405 National Renewable Energy Laboratory, 15013 Denver West Parkway, Golden, Colorado 80401, United States; § Advanced Energy Systems Graduate Program, 3557 Colorado School of Mines, Golden Colorado 80401, United States; ∥ Department of Chemistry, University of Puerto Rico Humacao Campus, Humacao 00792, Puerto Rico

**Keywords:** anion exchange membrane electrolysis, ionomer, NiO, IrO_2_, interface, oxygen evolution, degradation mechanisms

## Abstract

For anion exchange membrane (AEM) electrolysis to be cost- and performance-competitive to proton exchange membrane (PEM) electrolysis, evaluating and improving the stability of the ionomer at the ionomer–catalyst interface will be key to this emerging technology. Theoretical calculations of molecular fragments of the ionomers detailed the complex degradation mechanisms accessible to four different classes of ionomers (Nafion, Sustainion, Versogen, and quaternary ammonium types—ETFE, Gen 2, and Georgia Tech) on model catalysts of platinum group metal IrO_2_ and earth-abundant NiO. These mechanisms may occur during the making of the ionomer-catalyst ink or in the alkaline environment of AEM electrolysis or are energetically accessible at the electrochemical potentials of electrolysis. We identified diverse degradations such as (H)­SO_4_ production, water formation, oxidation to an alcohol, and deprotonation, leading to ionomer instability and competing with the oxygen evolution reaction (OER). Theory predicted that the weakly bound, intact cations of Sustainion’s methyl imidazolium on NiO and Versogen’s piperidinium on NiO combinations to be particularly stable and active for OER; these findings were validated by half-cell rotating disk electrode tests, where following break-in, their performance increased by 7–8 times. IrO_2_ may be stable and maintain OER activity, but site access remains limited due to the strong binding and reactivity of the ionomer at the high potentials of electrolysis (at 1.4 V, Nafion’s SO_3_ splits into SO_2_ + O; at 0.6 V, double deprotonation of Versogen can occur; at 1.5 V, ring oxidation of Sustainion to an alcohol initiates).

## Graphical abstract

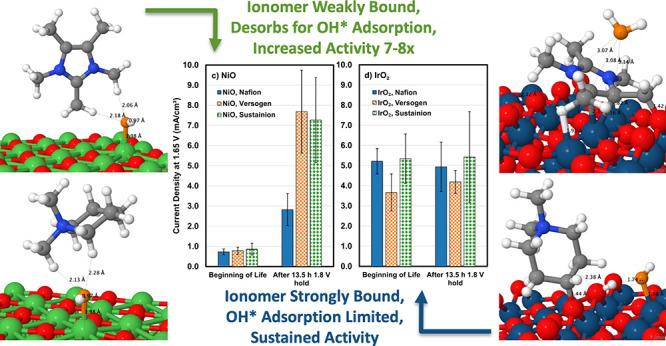

## 1 Introduction

Anion exchange membrane (AEM) electrolysis is an emerging technology for the renewable production of hydrogen when coupled with electrical sources of energy such as solar and wind, enabling the use of cheap, earth-abundant metals for the catalyst and other components of the membrane electrode assembly.^1,2^ However, earth-abundant metal catalysts may be sensitive to poisoning and degradation due to the high pH of the electrolyte, interactions with the AEM ionomer, or ionomer–electrolyte conductivity effects.^3–8^ While past studies have limited themselves to studying the ionomer in isolation,^9–16^ a small set of ionomers to a single catalyst,^4,17,18^ or a single ionomer to an array of catalysts,^17–20^ our study delves into detail the chemical degradation mechanisms available to the ionic fragments of 8 different ionomer–catalyst combinations: four different classes of ionomers (Nafion, Sustainion, Versogen, and quaternary ammonium types—ETFE, Gen 2, and Georgia Tech) on model catalysts of platinum group metal IrO_2_ and earth-abundant NiO. We detail the chemical degradation mechanisms present during the making of ionomer–catalyst inks and also in the presence of the hydroxide ion (the first step to the oxygen evolution reaction of electrolysis; the anion present in electrolytes utilized in the electrochemical cell).

The performance of AEM ionomers is often benchmarked against Nafion, Dupont’s perfluorinated sulfonic acid ionomer, which remains the high-performing proton exchange membrane (PEM) of choice in PEM fuel cells and electrolysis, maintaining chemical stability at the high potentials and long operating times of OER.^9,21^ Much of the experimental and theoretical work has focused on hydration levels and channel cluster models related to proton conductivity with degradation of the membrane attributed to the presence of trace radical species, morphological changes due to drying treatments, or back-pressure leading to cross-permeation of H_2_ and O_2_.^9–13^ Another consideration posed by Warren et al. is the possibility of sulfonic acid dissociation in the presence of water; Nagao et al. noted that hydrogen bonds orienting sulfonic acid groups into a rigid structure could also restrict proton conductivity.^22,23^ Nafion remains particularly susceptible to degradation in the presence of an OH radical or hydrogen peroxide, highlighting that the presence of contaminants in the electrolyte can also influence ionomer stability.^13,24^

This suggests that AEM ionomers may have similar issues in an alkaline electrolyte: there may be other chemical interactions at the interface that possibly contribute to the drops in activity, related to the ionomer-electrolyte (hydrogen bonds, OH^–^ adsorption to ionomer functional groups) or the ionomer-catalyst (side reactions leading to competing products).^25^ Li et al. coupled NMR kinetic analysis with density functional theory (DFT) calculations to showcase that the oxidation of the ionomer’s phenyl group contributed to AEM performance decay in Pt-, Ir-, and LaSrO-based materials.^5^ Ghoshal et al. noted that different membrane–ionomer combinations could lead to a change in the Tafel slope, implying that the ionomer could influence OER mechanisms and activity by suppressing the Co^3+^ to Co^4+^ transition (the desirable state for catalysis in commercial Co nanoparticles).^4^ Luo et al. found that the liquid-vapor uptake of AEM ionomers could vary considerably, leading to differences in hydroxide ion (OH^–^) mobility through a fuel cell and adverse effects of water-swelling on the mechanical stability of the ionomer.^15,16^ Chakraborti et al. noted that the Grotthus mechanism contributes to OH^–^ diffusion and that there can be a nonlinear dependence in hydration to diffusion in Sustainion.^14^ Krivina et al. focused on isolating electrolyte-ionomer interactions in their electrochemical tests with theoretical calculations offering insight into the highest occupied molecular orbitals of the ionomer fragment.^17^

However, these studies expose the lack of systematic understanding of the chemical interactions unique to the facet or crystalline form of different materials in contact with an ionomer that can lead to (in)­stability of the ionomer–catalyst interface. Moreover, the underlying chemistry of an ionomer in the presence of key OER intermediates may further reveal (in)­stability of the ionomer-catalyst in an alkaline environment and whether some catalysts may be more (or less) susceptible to competing reactions to OER, further leading to performance losses. In this joint theoretical–experimental study, plane-wave density functional theory (PW-DFT) calculations were performed to probe the binding strength of different functional groups from popular ionomers (Nafion, Sustainion, Versogen, and alkyl quaternary ammoniums—Gen 2, ETFE, Georgia Tech)^8,24,26–29^ to catalyst sites on benchmark materials such as NiO, an earth-abundant material, and IrO_2_, a platinum group metal material. NiO and IrO_2_ are model catalysts that are commercially available and known to be well-characterized and homogeneous: NiO in the rock-salt phase and IrO_2_ in the rutile phase, allowing for our theoretical model and experiments to match.^19,20,30^

This enabled us to understand the stability of the ionomer-catalyst interface and possible poisoning of active sites: if the functional group binds and reacts with the catalyst, then it may be unstable and suffer degradation; if it binds more strongly than key reaction intermediates such as OH* (the first step to the oxygen evolution reaction—OER), then the ionomer effectively poisons the catalyst by blocking active sites. Furthermore, coadsorption of the ionomer with OH* allowed us to evaluate the stability and activity of the ionomer in consideration of the alkaline environment and in the context of OER activity. These calculations were coupled with rotating disk electrode (RDE) tests to evaluate the ionomer–catalyst performance of commercially available NiO and IrO_2_.

## 2 Results and Discussion

Nafion is a well-known benchmark membrane typically used in proton exchange membrane fuel cells and electrolyzers. It is also utilized by experimentalists to compare to newly developed AEM ionomers such as Sustainion, PiperION, and alkyl quaternary ammoniums types such as Gen 2, ETFE, Georgia Tech (see Figure 1).^8,24,26–29^ In sections 2.1–2.4, we highlight adsorption trends of the ionomer–catalyst alone and the ionomer–OH interactions on benchmark catalysts NiO and IrO_2_. Ideally, the ionomer remains intact when in contact with the catalyst and binds more weakly than OH*, allowing OH* to adsorb to metal active sites. Li et al. highlighted phenyl oxidation to an alcohol to be a degradation mechanism on Pt- and Ir-based catalysts; Yu et al. identified the H^•^ radical to be of greater importance in breaking off the sulfonic acid group to form H_2_SO_3_ or SO_2_ and the susceptibility of Nafion degradation in the presence of OH on Pt catalysts.^5^ In this paper, theory and experiment found that different ionomer-catalyst combinations could yield, unsurprisingly, different measures of stability and activity.

**Figure 1:**
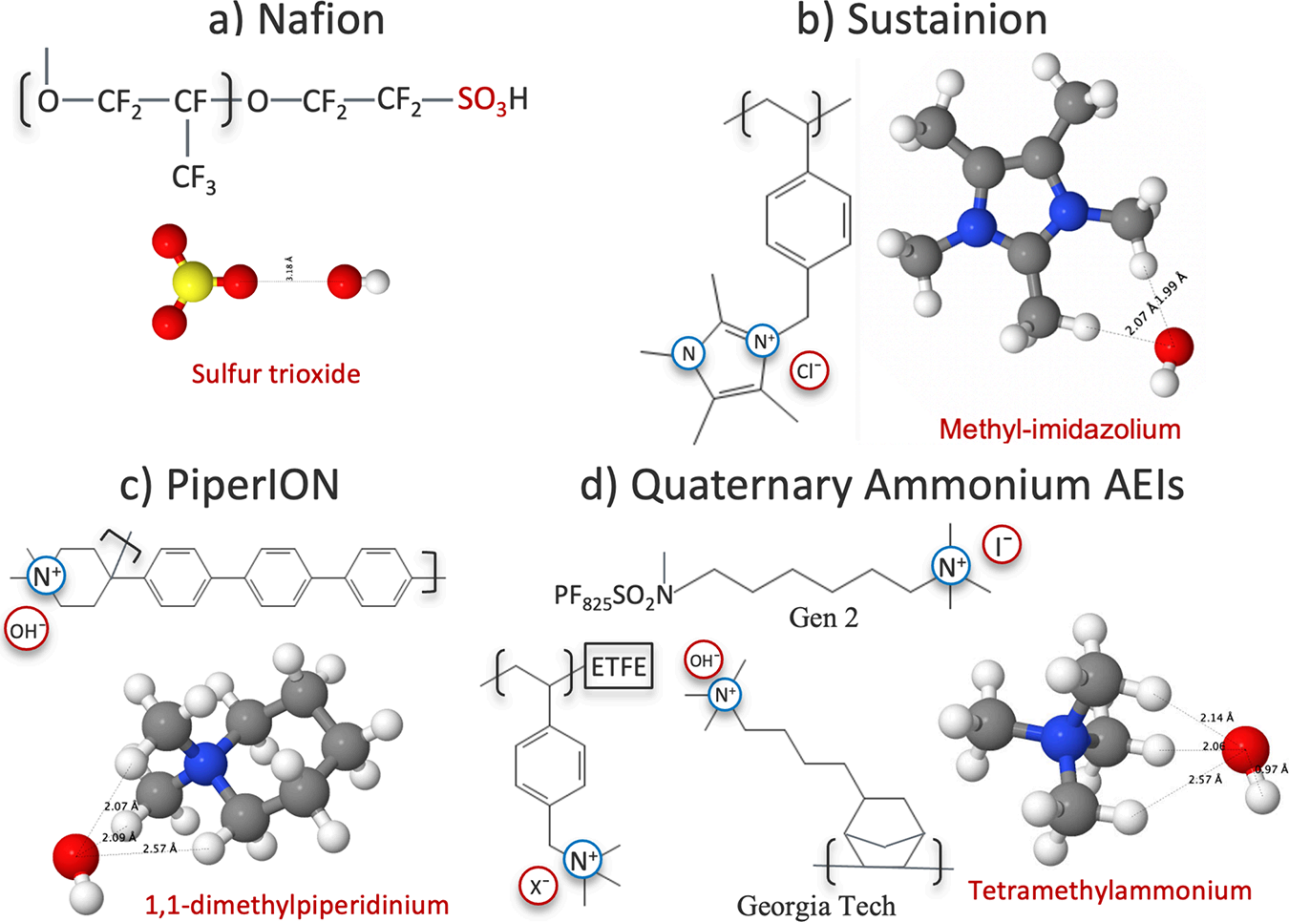
Four ionomer fragments of (a) sulfur trioxide (representing the anion for Nafion), (b) 1,3,4,5-tetramethyl-2,3-dihydro-1*H*-imidazol-1-ium or methylimidazolium for brevity (representing the cation for Sustainion), (c) 1,1-dimethylpiperidinium (representing the cation for PiperION), and (d) tetramethylammonium (representing the cation for alkyl quaternary ammonium anion exchange ionomers—AEIs: Gen 2, ETFE, and Georgia Tech).

PW-DFT calculations identified a considerable range of degradation mechanisms including deprotonation, water formation, alcohol production, demethylation, or (bi)­sulfate creation depending on the ionomer/catalyst combination. Much of the theoretical focus in previous studies has been degradation mechanisms of the ionomer alone or on PGM catalysts.^5,13^ While the benchmark, non-PGM NiO catalyst is significantly less active than IrO_2_ for OER, this relatively inactive catalyst introduced numerous degradation mechanisms in AEM ionomers. Moreover, there may be “rarer”, higher energy interactions in which these ionomer fragments become reactive at the high potentials of OER related to what previous studies have attributed to radical formation, morphological changes, or cross-permeation of gas streams.^9–13^ Greater understanding of AEM ionomer stability at an atomic and electronic resolution can guide and enable experiment to improve ionomer/catalyst combinations to achieve operation comparable or greater than that of PEM electrolysis. In section 2.5, half-cell rotating disk electrode (RDE) experiments examined the stability and activity of Nafion, Sustainion, and Versogen on NiO and IrO_2_ to compare to theoretical trends found in sections 2.1–2.4.

### 2.1 Nafion

While Nafion was initially developed for use in PEM fuel cells, its stability under high oxidative potentials and its superior ionic conductivity in PEM has allowed it to be a basis of comparison for AEM electrolysis.^6,9,31,32^ In the alkaline environment of AEM electrolysis, the deprotonation of the −HSO_3_ functional group by a hydroxide ion would be expected and, therefore, theoretical calculations focused on interactions of the sulfur trioxide group (SO_3_) on the NiO (Figure 1a,b) and IrO_2_ (Figure 1c,d) catalysts. The sulfur trioxide can potentially block up to two metal active sites on both NiO and IrO_2_: it adsorbs more strongly on NiO than OH* and acts as a poison to this catalyst; it adsorbs more weakly on IrO_2_ than OH* but can also block a combination of surface Ir (Ir_5f_) and bridging O (O_b_) atoms (Iso II), which can halt one pathway to deprotonation. In our previous mechanistic study of the different pathways to OER, the bridging O of IrO_2_ can help deprotonate OH* with an activation energy of 0.18–0.41 eV, depending on the surface coverage of O*/OH*.^33^ SO_3_ isomers bind more strongly on IrO_2_ with an associated greater charge transfer from the surface to this functional group of up to +1 e. The sulfur atom of SO_3_ can pull charge from surface Ir, causing Ir to be circa +1.3 to +1.4 e, whereas the O atom of SO_3_ results in surface Ir being circa +1.6 e.

Marković et al. noted a drop in Pt’s activity when RDE studies were performed in sulfuric acid as compared to perchloric acid, attributing this to the inhibiting effect of (bi)­sulfate anions adsorbed to the surface.^34,35^ This suggests that greater attention must be paid to how the ionic functional groups of ionomers may influence the catalysis of different materials, including PGMs and non-PGMs. Moreover, Danilovic et al. noted that the onset of OER activity at +1.23 V often coincided with the dissolution of Ru- and Ir-based catalysts, attributing the instability of these RuO_*x*_, IrO_*x*_ materials to their transition to higher oxidation states from *n* = 4+ to *n* > 4+.^36^ Our theoretical calculations indicate that at potentials of 0.9 V and beyond, SO_3_ can activate a dissolution mechanism on IrO_2_ where the bridging oxygen can be removed and replaced by SO_3_ (Iso. IV) or Nafion’s SO_3_ can degrade to SO_2_* + O* (Iso. VI).

In the alkaline environment of AEM electrolysis, OH* can potentially react with SO_3_. On NiO, without OH*, the SO_3_ functional group remained whole but poisoned metal active sites. However, once OH* was introduced to the unique geometries of isomers I, III, and V, the sulfate ion (SO_4_^2–^, iso I) and hydrogen sulfate ion (HSO_4_^–^, iso II–iso IV) spontaneously formed with appropriate charge transfer for these ions of circa −2 e and −1 e. Therefore, theory predicted that the ionomer-catalyst combination of Nafion–NiO will initially suffer performance loss due to the poisoning of Ni sites by Nafion and competing reactions to form SO_4_^2–^ and HSO_4_^–^ instead of OER. However, at potentials of >0.7 V, stable OH* adsorption occurs and OER may continue. Experimental potentials range between 1.6 and 2.0 V for electrolysis, allowing for SO_3_ to possibly desorb from the surface (Figure 2a, isomers II–IV) or access stable coadsorption of SO_3_* and OH* (Figure 2b, isomers V–VI). In comparison, since Nafion was developed and optimized to work with PGM catalysts, it remained intact in the presence of OH* on IrO_2_. SO_3_ can potentially block two Ir sites or one Ir, one O_b_ site but OH* could adsorb to neighboring Ir sites and was even stabilized by hydrogen bonds to the O atoms of SO_3_.

**Figure 2:**
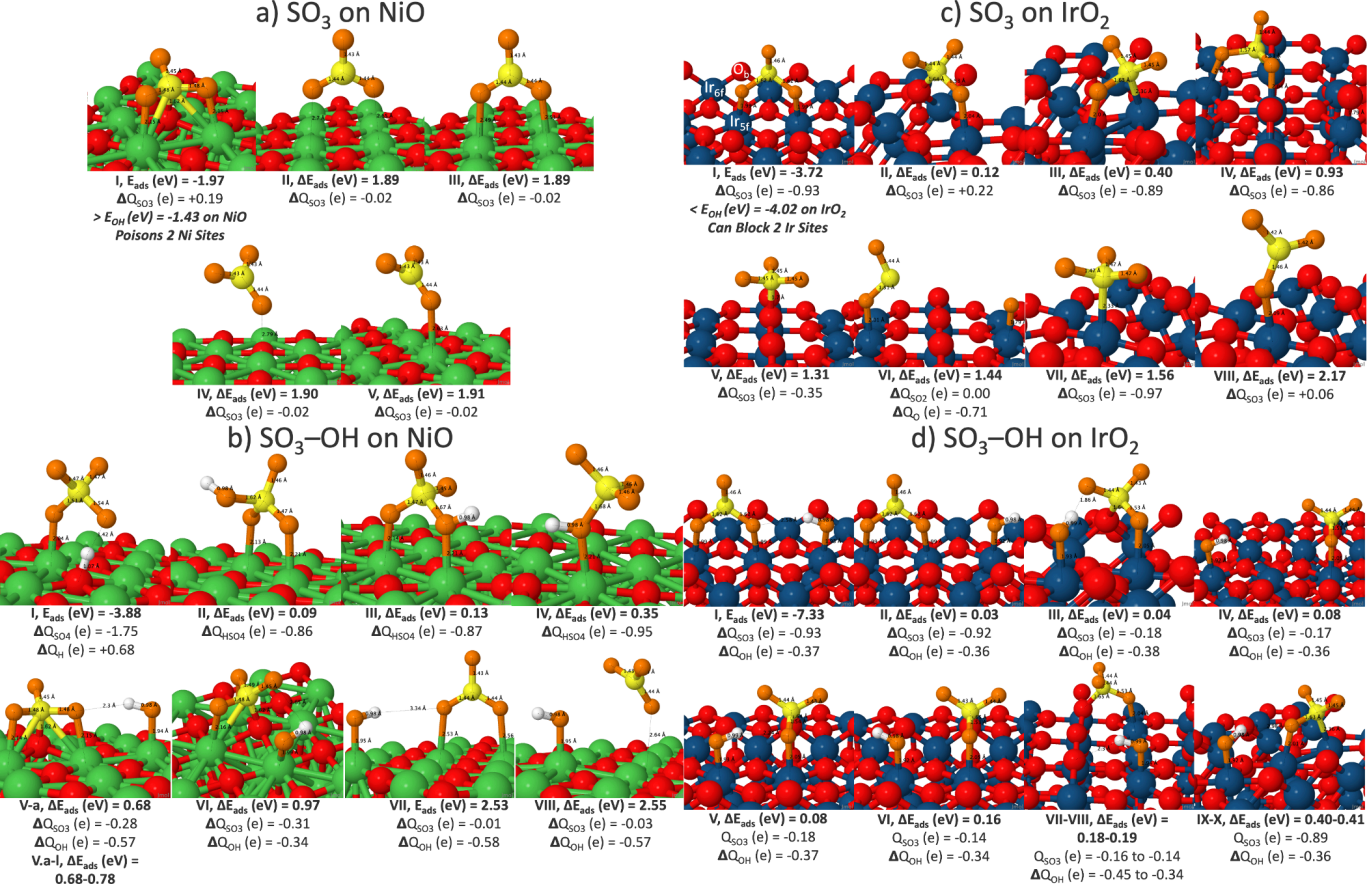
Nafion’s SO_3_ and SO_3_–OH isomers on (a, b) NiO (100) and (c, d) IrO_2_ (110) with Bader charges (Δ*Q*), bond distances (Å), adsorption energy (*E*_ads_), and adsorption energy relative to the global minimum (Δ*E*_ads_). Ni atoms in green, surface O in red, Ir in teal, H in white, S in yellow, and adsorbate O in orange. IrO_2_’s active sites include the 5-fold coordinated Ir_5f_ and bridging oxygens (O_b_, topmost oxygens coordinated to two six-fold coordinated Ir atoms, Ir_6f_).

### 2.2 Sustainion

Sustainion is classified as an AEM ionomer with a polystyrene backbone and a tetramethylimidazolium-based cation for transporting OH to the anode for the half-cell reaction, OER.^4^ A product of Dioxide Materials, Inc., Sustainion can potentially reach performance of 1 A/cm^2^ at circa 1.9 V, maintaining stability across 11,000 cycles during accelerated voltage shock tests.^37–39^ Moreover, Sustainion’s stability and high performance has translated well into CO_2_ electrolysis for CO selectivity of >98% during six months of operation for formic acid production, depending on the catalyst and electrochemical cell’s configuration.^26^

For brevity, we refer to tetramethylimidazolium as imidazolium in the following text. Imidazolium binds weakly to NiO at −0.69 eV and remains unreactive in the presence of OH, physisorbing via hydrogen bonds and validating its commercial availability as a stable AEM ionomer (Figure 3a). Since imidazolium could adsorb in a variety of geometries (planar or perpendicular to the surface, monodentate or bidentate with different −C–CH_3_, −N–CH_3_ functional groups) on NiO, we coadsorbed OH* on all nonequivalent Ni sites of isomers I–IV and VI. Imidazolium often retains its circa +1 e ionic character when adsorbed on NiO or as a cation–anion complex with OH*. For OH to remain attached to imidazolium, these states are 0.7 eV higher in energy. The binding strengths of imidazolium* alone and imidazolium-OH* were nearly comparable at circa −0.7 eV, showcasing that Sustainion may potentially add repulsive, steric effects to the ionomer-OH-catalyst interface (Figure 3b).

**Figure 3:**
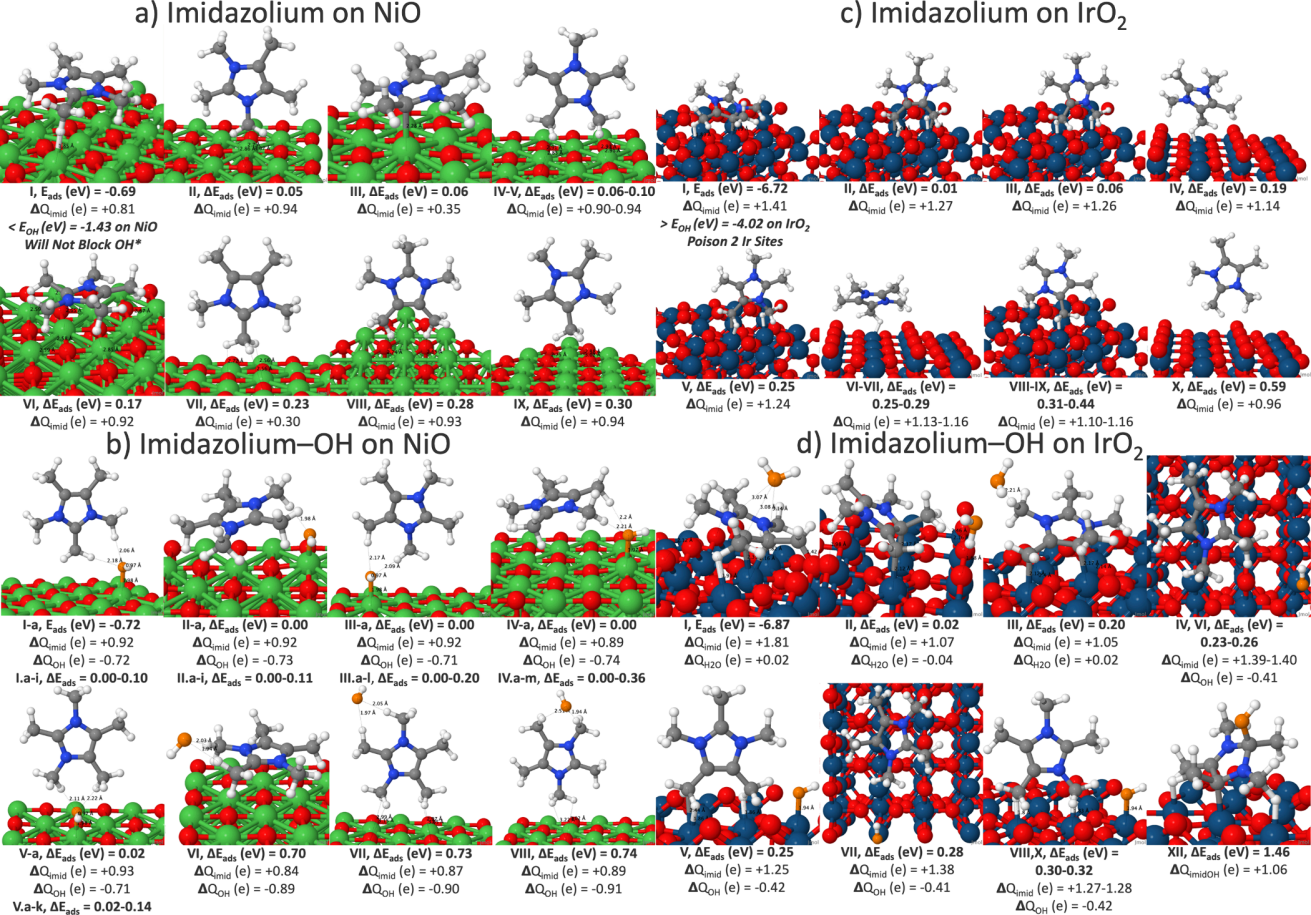
Sustainion’s imidazolium and imidazolium-OH isomers on (a, b) NiO (100) and (c, d) IrO_2_ (110) with Bader charges (Δ*Q*), bond distances (Å), adsorption energy (*E*_ads_), and adsorption energy relative to the global minimum (Δ*E*_ads_). Ni atoms in green, surface O in red, Ir in teal, H in white, N in blue, C in gray, and adsorbate O in orange.

In contrast to NiO, imidazolium adsorbs more strongly than OH* on IrO_2_ with corresponding charge transfer >+1 e and poisons up to two surface Ir sites (Figure 3c). Similar to NiO, the imidazolium remains whole on IrO_2_, exhibiting a variety of possible geometries on IrO_2_ and theoretical calculations accounted for coadsorption of OH* on isomers I–IV. Upon coadsorption with OH*, the OH* deprotonates imidazolium to form water on the planar configuration of imidazolium (Figure 3d, isomers I–III). The planar imidazolium’s −C–CH_3_ functional group easily sheds its H to OH to form water with the −C–CH_2_ attaching to a surface Ir atom. Moreover, the planar configuration may further react at the higher operating potential of circa 1.5 V to oxidize into an alcohol. Contrary to the planar configuration, the imidazolium oriented perpendicular to the surface is unreactive with these intact, stable coadsorbed OH* + imidazolium* isomers being >0.2 eV higher in energy (isomers IV–X).

These theoretical calculations indicate that the ionomer-catalyst combination of Sustainion-IrO_2_ may easily degrade due to deprotonation to form water over OH* adsorption, the key first step to OER, or oxidize into an alcohol at circa 1.5 V. In our previous, in-depth mechanistic study of OER on IrO_2_ (110), we observed that this surface spontaneously splits water and remains highly reactive to OER.^33^ However, the high reactivity of the imidazolium fragment to OH* may reflect Sustainion’s instability in alkaline environments as the planar configuration (isomers I–III, XII) continues to react with OH. We note that Sustainion employs additional phenyl rings and the polystyrene backbone to potentially mitigate the reactivity of the imidazolium: there may be additional steric effects that prevent the cation from adsorbing in the planar configuration.

### 2.3 Versogen

Versogen’s poly­(aryl piperidinium) based on terphenyl (W7, PAP-TP-85, or PiperION) AEM ionomer previously demonstrated high stability and activity in various benchmarking studies focused on commercial catalysts, water uptake, and techniques for quantifying polarization resistance.^6,7,16,31,40^ It also exhibited high ionic mobility in hydroxide-exchange membrane fuel cells and CO_2_ electrolyzers, validating its commercial viability across multiple technologies.^16,17^ However, Lindquist et al. observed degradation of the PiperION membrane for AEM electrolysis following 175 h of steady-state operation with X-ray photoelectron spectroscopy detecting oxidized species and F/N loss.^18^

Theoretical calculations focused on the 1,1-dimethylpiperidinium ion fragment of Versogen, which we will refer to as piperidinium for brevity. Piperidinium binds weakly to the NiO surface and remains intact. Similar to Sustainion’s imidazolium, piperidinium can adsorb in different orientations, many of which are close in energy of <0.20 eV (Figure 4a). Therefore, coadsorption of OH* occurred on the more distinct isomers I, II, IV, and V (isomer III being close to a tilted isomer I). Upon coadsorption with OH*, these unique piperidinium-OH* geometries are nearly degenerate with a relative energy of 0.02 eV. In Figure 4b, we summarize the most stable, unique piperidinium-OH* geometries and details of the other Ni-OH* sites possible for the same piperidinium geometry may be found in the Supporting Information. Piperidinium prefers to hydrogen-bond to the adsorbed OH* rather than covering the Ni active sites, allowing OH* to readily access Ni sites and the isomer configurations (V–VIII) in which OH adsorbs to the piperidinium ion rather than a Ni site are circa 0.7–0.9 eV higher in energy. In conclusion, theory predicts that the ionomer-catalyst combination of Versogen-NiO will be stable in the alkaline environment of electrolysis and facilitate the first step of OER, OH* adsorption.

**Figure 4:**
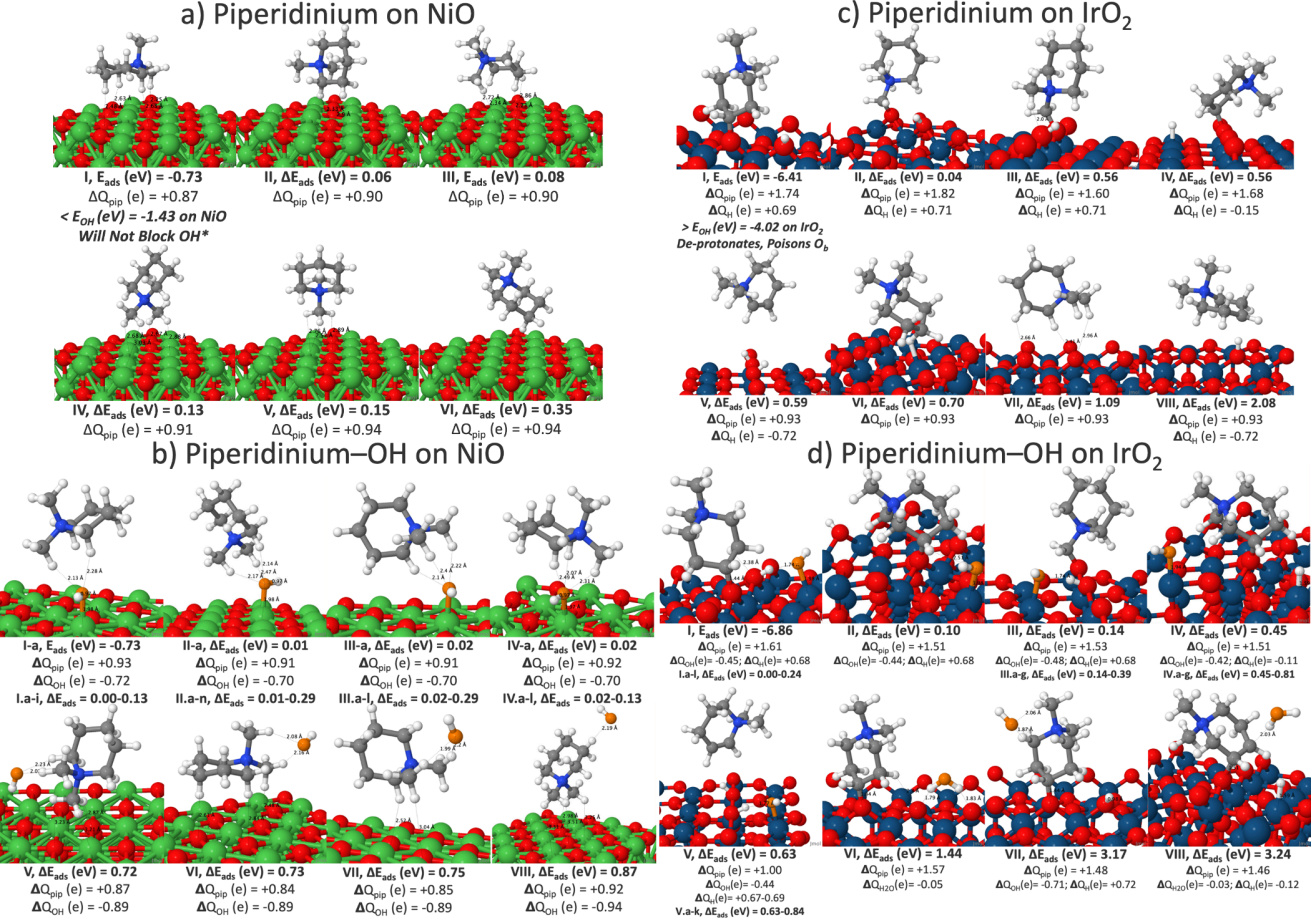
Versogen’s piperidinium and piperidinium-OH isomers on (a, b) NiO (100) and (c, d) IrO_2_ (110) with Bader charges (Δ*Q*), bond distances (Å), adsorption energy (*E*_ads_), and adsorption energy relative to the global minimum (Δ*E*_ads_). Ni atoms in green, surface O in red, Ir in teal, H in white, N in blue, C in gray, and adsorbate O in orange.

In contrast to piperidinium on NiO, piperidinium on IrO_2_ adsorbs more strongly than OH*, poisoning surface Ir/O sites, and degrades via deprotonation (Figure 4c). Piperidinium tends to deprotonate onto bridging oxygens, either from its ring hydrogens (Isomers I, IV, V, and VIII) or its methyl groups (Isomer II). We note that for isomers I, IV, and V, the C–H bonds opposite of the nitrogen would typically be attached to long benzene rings but can still represent a possible route to degradation since radicals, contaminants, the high potentials of OER, or hydroxide ions can potentially facilitate scission of C–C, C–N, and C–H bonds to fracture aromatic rings or the cation functional group.^13^ At a moderate potential of 0.6 V, piperidinium may also double deprotonate from its ring (Isomer V). The second step to OER, OH splitting, occurs via deprotonation onto bridging oxygens (Ir_5f_OH* → Ir_5f_O* + O_b_H*), suggesting that this step could be affected if piperidinium continues to hydroxylate or bind via the aromatic ring’s −CH onto available bridging oxygens. The intact piperidinium appears at circa 0.6 V, but theory predicts that the deprotonated piperidinium will dominate on the surface and occupy various surface Ir_5f_ and O_b_ sites.

Upon coadsorption of OH* with isomers I, II, IV, and V, OH often hydrogen bonds to the H* on bridging oxygens or the piperidinium’s hydrogens (Figure 4d). At circa 1.4 V, water formation occurs, suggesting that at the high operating potentials of OER, Versogen will also introduce competing reactions to OER. This exacerbates the known stability issues related to PGM-catalysts for OER at higher potentials.^18,36^ Lindquist et al. observed that after an initial break-in of 20 h where benchmarked AEM ionomers (Aemion, Sustainion, PiperION) all exhibited degradation rates of 11–15 mV h^–1^, Versogen’s PiperION subsequently endured with a reduced degradation rate of 0.67 mV h^–1^.^18^ Our theoretical calculations indicate that the dominant product for Sustainion would be water formation, in direct competition to OER, whereas Versogen’s PiperION allows for unreacted, coadsorption of piperidinium and OH* up to 1.4 V. This may explain the reduced degradation rate exhibited by PiperION following the 20 h break-in period. Indeed, in our previous study on the mechanistic pathways available to IrO_2_ (110) for deprotonation or O_2_ formation, the activation energies of these pathways could vary by up to 0.2 eV depending on the surface O/OH coverage.^33^ The more complex environment of the ionomer-OH-catalyst interface may similarly contribute to changes to the degradation rate following initial operation.

### 2.4 Alkyl Quaternary Ammonium AEM Ionomers: ETFE, Gen 2, Georgia Tech

ETFE (ethylene tetrafluoroethylene), Gen 2 (perfluorinated AEM ionomer developed at NREL), and Georgia Tech (university collaborator) represent a class of AEM ionomers utilizing the alkyl quaternary ammonium for OH^–^ ion exchange.^8,27,28,41^ This cation allows for a compact ion exchanger, whose additional alkyl chains provide greater stability and lower molecular mass compared to perfluorinated and polyaromatic AEM ionomers.^8^ These large ionomers are simplified to focus on the cation fragment tetramethylammonium to probe stability on the non-PGM NiO and PGM IrO_2_ surface should this ion come into contact with the catalyst.

PW-DFT calculations found that the tetramethylammonium cation spontaneously dissociates into component groups, trimethylamine, N­(CH_3_)_3_, and the methyl group, CH_3_, on NiO (Figure 5a). In the presence of an adsorbed OH*, OH* can further react with the trimethylamine group, resulting in the formation of H_2_O and the reattachment of a methyl group to the trimethylamine via a C–C bond (isomer I, Figure 5b). The second possible competing reaction to OER is methanol (CH_3_OH) formation (isomers II–V) from the OH attaching to the methyl group, indicating that these ionomers may introduce competing reactions to OER. The isomers in which OH* remains intact to adsorb to a Ni site and remain available for OER are 0.7–0.9 eV higher in energy. Our calculations may explain in part the degradation mechanisms possible for these alkyl quaternary ammoniums ionomers—the NiO surface is not particularly active, yet the tetramethylammonium will dissociate both on the clean surface and in the presence of an adsorbed OH*. The ionomer-catalyst combination of tetramethylammonium-NiO will exhibit reduced OER activity and even introduce impurities to the electrolyte as the methanol product desorbs from the surface.

**Figure 5:**
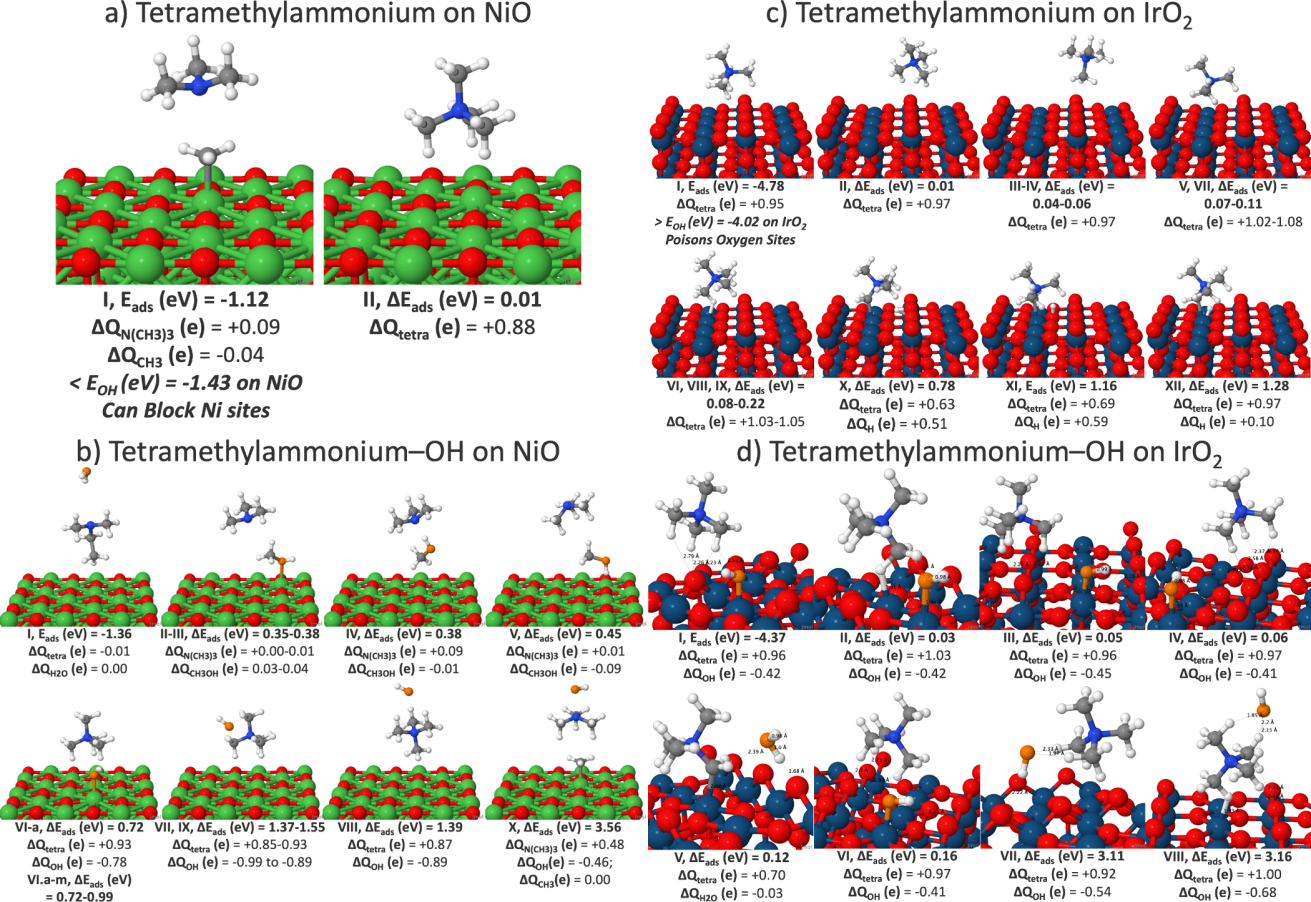
ETFE, Gen 2, and Georgia Tech’s tetramethylammonium and tetramethylammonium-OH isomers on (a, b) NiO (100) and (c, d) IrO_2_ (110) with Bader charges (Δ*Q*), bond distances (Å), adsorption energy (*E*_ads_), and adsorption energy relative to the global minimum (Δ*E*_ads_). Ni atoms in green, surface O in red, Ir in teal, H in white, N in blue, C in gray, and adsorbate O in orange.

Compared to NiO, quaternary ammonium-based ionomers may suffer durability issues on IrO_2_ at potentials >0.7 V. At this moderate potential, spontaneous deprotonation occurs with hydrogen atoms attaching to bridging or surface oxygen, iridium sites (Isomers X–XIII). Many of the isomers adsorb more strongly than OH* (isomers I–IX), isomers I–IV block O_b_ sites, and isomers VI, VIII, and IX poison an Ir site. Theoretical calculations considered coadsorption of OH* with tetramethylammonium in the tridentate, bidentate, and monodentate positions (isomers I–III) as well as in its more activated geometry of Ir_5f_ coordinated to the adsorbate H (isomer IV). While tetramethylammonium allows for stable coadsorption of OH*, at a low potential of >0.12 V, water formation occurs. On both NiO and IrO_2_, theory predicts that this class of ionomers may be unstable and degrade at potentials of >0.8 V with degradation arising through either demethylation (on NiO) or deprotonation (on IrO_2_). Moreover, these ionomers will introduce competing reactions to OER such as methanol (Figure 5b).

Therefore, in the following section, experiments focused on the ionomer-catalyst performance of the more stable, commercially available ionomers Nafion, Sustainion, and Versogen on benchmark catalysts, NiO and IrO_2_. In Table 1, theoretical predictions of the possible degradation mechanisms available at varying potentials to different ionomer-catalyst combinations are summarized. NiO-Sustainion and NiO-Versogen catalyst-ionomer combinations are predicted to be the most stable and active, since they preferentially allow OH* adsorption on Ni active sites; IrO_2_-Nafion and IrO_2_-Sustainion may potentially be more advantageous for OER since they are susceptible to a limited range of degradation mechanisms. The availability of degradation mechanisms at the varying potentials of electrolysis may be indicative of lower-performing (NiO-Nafion; IrO_2_-Versogen) and higher-performing (NiO-Sustainion, NiO-Versogen) catalyst-ionomer combinations. In particular, how strongly the ionomer binds to the catalyst may determine the availability of metal active sites: the nonpolar NiO may benefit from the hydroxide ion conductivity of the ionomer and easily recover active sites due to the weakly bound ionomer (less than −1 eV); in contrast, IrO_2_’s active sites are set during the ionomer-catalyst ink creation due to how strongly bound the ionomer is (more than −3 eV). Volk et al. suggested that the ionomer may also act more as a binder, minimizing catalyst delamination and determining the best performance to occur with catalyst inks made with 10 wt % Versogen or 10 wt % Nafion.^42^

**Table 1:** (In)­stability Trends of Ionomer-Catalyst versus Ionomer-OH-Catalyst Interface Predicted by Theory

	Ionomer-NiO	Ionomer-OH*-NiO	Ionomer-IrO_2_	Ionomer-OH*-IrO_2_
Nafion	Unreactive	At 0 V, SO_4_* + H*	At 0 V, Unreactive	Unreactive coadsorption
		Sulfate Creation	At 1.44 V, SO_2_* + O*	
		At 0.09 V, HSO_4_*	SO_3_ degradation	
		Sulfuric Acid Creation		
		At 0.68 V, Unreactive coadsorption for OER		
Sustainion	Unreactive	Unreactive co-adsorption for OER	Unreactive	At 0 V, C_8_H_14_N_2_* + H_2_O*
				At 0.23 V, Unreactive co-adsorption
				At 1.5 V, C_8_H_15_N_2_OH*
				Ring oxidation to alcohol
Versogen	Unreactive	Unreactive coadsorption for OER	At 0.00–0.56 V, C_7_H_15_N* + H*	At 0 V, C_7_H_15_N* + H* + OH*
			Deprotonation	Unreactive coadsorption
			At 0.59 V, C_7_H_14_N* + 2H*	At 1.44 V, C_7_H_15_N* + H_**2**_ O*
			Double deprotonation	Water Formation
Tetramethylammonium	At 0 V, N(CH_3_)_3_ + CH_3_	At 0 V, N(CH_3_)_2_CH_2_CH_3_ + H_2_O	Unreactive	At 0 V, Unreactive coadsorption
	Demethylation	Water Formation		At 0.12 V, N(CH_3_)_3_CH_2_* + H_2_O
	At 0.01 V, Unreactive			Water Formation
		At >0.35 V, N(CH_3_)_3_ + CH_3_OH		
		Methanol Production		
		At >0.7 V, Unreactive coadsorption with OH*		

### 2.5 Experimental Results

The initial activities for two OER catalysts (NiO and IrO_2_) were evaluated using half-cell RDE testing, with the results shown as Tafel plots in Figure 6a,b. RDE avoids the complications of device-level testing (materials integration, ink rheology, and electrode structure) and allows for a focused assessment of kinetic trends by isolating kinetic performance from contributions related to solution resistance and mass transport.^31^ RDE test conditions have complications, and a nontrivial understanding is needed in leveraging half-cell testing to support simulations and in using half-cell testing as a proxy for single-cell, membrane electrode assembly (MEA) durability testing. These results are based on historical efforts in acidic and alkaline oxygen evolution that establish linkages and cautions when comparing modeling, RDE testing, and MEA testing.^20,43–47^ General guidance in RDE/MEA durability comparisons can be summarized as RDE can dramatically accelerate dissolution and oxidation processes (catalyst and ionomer), while underestimating the impact of catalyst layer properties on cell- and stack-level degradation, including ion/electron transport, suboptimal catalyst utilization, and complications from manufacturing or catalyst layer defects.^44,48^

**Figure 6:**
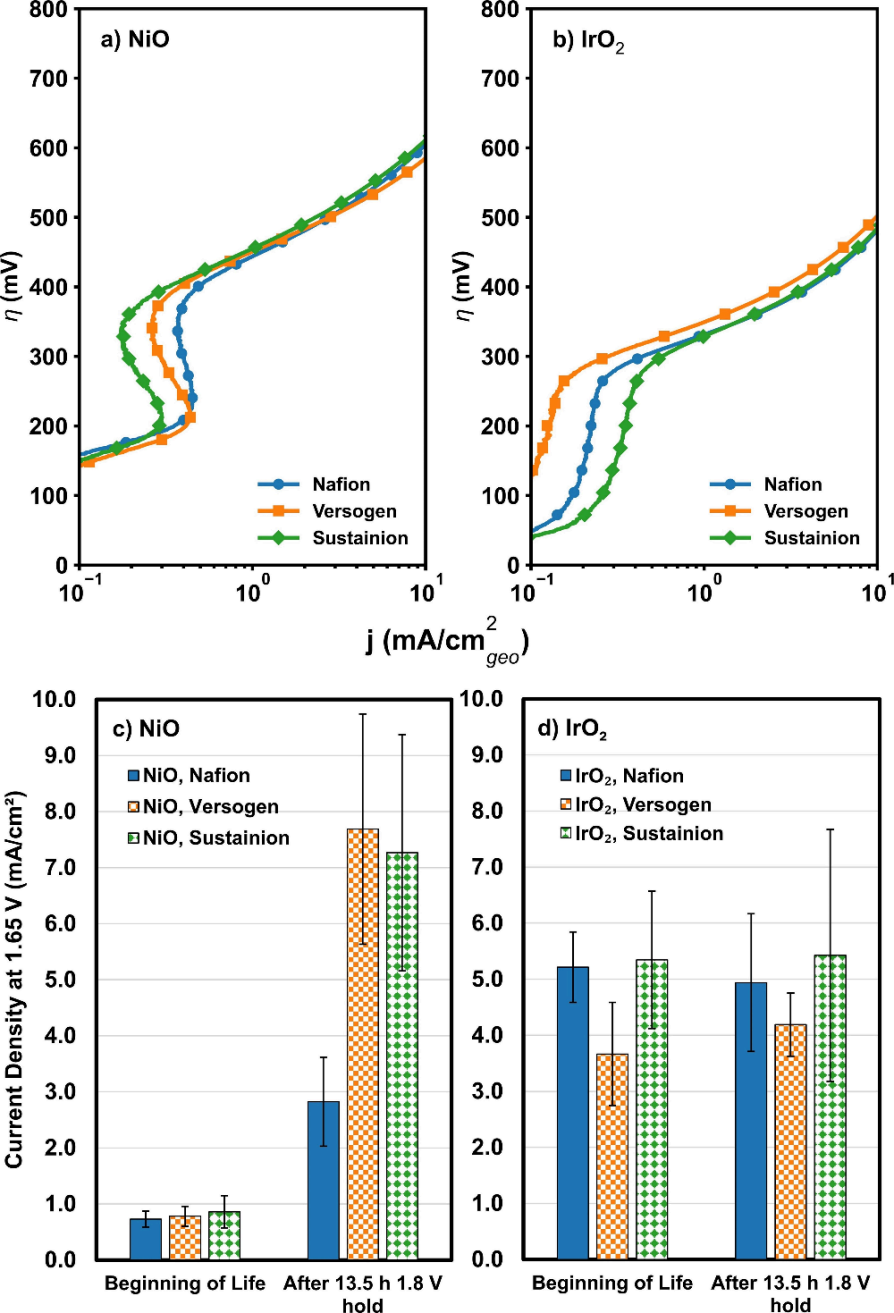
Tafel plots of initial catalytic activity for (a) NiO and (b) IrO_2_ and durability testing results for (c) NiO and (d) IrO_2_ with Nafion, Versogen, and Sustainion polymers. In (c) and (d), the results are shown as the average and standard deviation of three experiments.

For NiO, the initial electrochemical performance trends were similar for all polymer types. As shown in Figure 6a, NiO-Nafion (blue circles), NiO-Versogen (orange squares), and NiO-Sustainion (green diamonds) all had similar overpotentials across current density regimes. This can be further seen in the beginning of life current densities at 1.65 V for these materials (shown in Figure 6c), where the current densities were 0.7 ± 0.1 mA/cm^2^ (NiO-Nafion), 0.8 ± 0.2 mA/cm^2^ (NiO-Versogen), and 0.9 ± 0.3 mA/cm^2^ (NiO-Sustainion). NiO-Nafion, however, exhibited a higher Tafel slope (124 ± 13 mV/dec) than the NiO-Versogen and NiO-Sustainion samples (106 ± 1 and 113 ± 6 mV/dec, respectively; Table 2). A higher Tafel slope can be linked to changes in the electrochemical mechanism^49^ and is consistent with the theory expectation that NiO-Nafion kinetics may have been inhibited by competitive (bi)­sulfate formation side reactions.

**Table 2:** RDE Results of the Current Density at 1.65 V and the Tafel Slopes for NiO and IrO_2_ with Nafion, Versogen, and Sustainion Polymers before and after Durability Testing

		Current Density at 1.65 V (mA/cm^2^)		Tafel Slope (mV/dec)	
		Initial	After durability	Initial	After durability
NiO	Nafion	0.7 ± 0.1	2.8 ± 0.8	124 ± 13	86 ± 17
	Versogen	0.8 ± 0.2	7.7 ± 2.1	106 ± 1	79 ± 2
	Sustainion	0.9 ± 0.3	7.3 ± 2.1	113 ± 6	79 ± 5
IrO_2_	Nafion	5.2 ± 0.6	4.9 ± 1.2	92 ± 3	84 ± 4
	Versogen	3.7 ± 0.9	4.2 ± 0.6	94 ± 3	81 ± 3
	Sustainion	5.3 ± 1.2	5.4 ± 2.2	103 ± 9	86 ± 5

For IrO_2_, the initial electrochemical performance trends were also similar regardless of which ionomer was added to the catalyst: IrO_2_-Nafion, IrO_2_-Versogen, and IrO_2_-Sustainion all exhibited similar overpotentials across current density regimes as well as had similar Tafel behavior. The samples with IrO_2_ exhibited lower overpotentials (higher activities) than the NiO samples, consistent with literature expectations.^20,31,50^ These trends can also be seen clearly in the beginning-of-life current densities at 1.65 V, which were 5.2 ± 0.6, 3.7 ± 0.9, and 5.3 ± 1.2 mA/cm^2^ for IrO_2_-Nafion, IrO_2_-Versogen, and IrO_2_-Sustainon, respectively (Table 2).

Each catalyst-ionomer combination was further evaluated after chronoamperometry at 1.8 V (13.5 h) and the results were compared to initial performance trends. Performance trends were evaluated as the change in current density at 1.65 V, which was the kinetic region for all catalyst-ionomer pairs assessed, as was done previously.^31^ These results are shown in Figure 6c,d.

For the NiO samples, in all cases the electrochemical activities increased significantly (+200–900 % increase in current density at 1.65 V) after stability testing (Figure 6c). Theoretical calculations predicted that the Sustainion and Versogen ionomers’ cation would be bound weakly compared to OH* and remain stable on NiO. In contrast, Nafion’s SO_3_ binds more strongly than OH* and can potentially react with OH to form (H)­SO_4_. These binding trends related to OER performance may correlate more closely to experimental trends from durability testing. At high potentials of >1.8 V, SO_3_ can desorb from NiO, possibly halting the competing reactions of (H)­SO_4_ (see Figure 2a). Other experimental studies beyond this current work have observed that following initial break-in, ionomer-catalyst performance can improve significantly, suggesting that there may be more complex ionomer-catalyst interactions beyond the initial step of OER, OH* adsorption, and will be the subject of future mechanistic studies.^17,18,31^

Moreover, the performance of Ni oxide-based catalysts may be related to the synthesis and crystalline form of these materials beyond commercial, rock-salt NiO. The activation of Ni oxides towards OER via electrochemical aging has been reported previously for Ni films in alkaline electrolytes and attributed to a transformation from α-Ni­(OH)_2_/γ-NiOOH to β-Ni­(OH)_2_/β-NiOOH.^50–52^ Louie and Bell observed through in-situ electrochemical Raman spectroscopy investigation an increase in OER performance concurrent with this phase transformation and an accompanying anodic shift in the Ni II/III redox transition related to a decrease in the average Ni oxidation state.^52^

At 1.65 V, the current density of NiO-Nafion increased by +283 ± 41%; for NiO-Versogen and -Sustainion, this dramatically increased to +884 ± 84% and +756 ± 48%, respectively. This increased performance may be due to the desorption of the ionomer, allowing for OER intermediates to access Ni sites: Sustainion and Versogen physisorb at circa −0.7 eV whereas Nafion’s SO_3_ adsorbs more strongly at −1.97 eV. For all NiO samples, there was a decrease in the Tafel slope after testing; for all three polymers, the post-test Tafel slopes for all NiO samples were approximately 80–90 mV/dec, compared to 110–120 mV at beginning of life (Figure S13a, SI). This result indicates a possible change in the kinetics associated with either an activated crystalline form of NiO­(H) or that Versogen and Sustainion may be influencing other steps of OER beyond OH* adsorption. These experimental results highlight that the Versogen and Sustainion polymers may facilitate the electrochemical activation of NiO over Nafion, potentially validating theoretical results identifying SO_3_, (H)­SO_4_ blocking Ni active sites. Nafion potentially offers benefits to non-PGM catalysts as a binder, preventing catalyst detachment and allowing for significant performance enhancements by increasing the stability of the catalyst.^42^ Future work will delve more deeply into the effect of the ionomer on the 4-step mechanism to OER.

For all ionomer-IrO_2_ combinations, the electrochemical activities did not change significantly after durability testing (Figure 6d). There was, however, a decrease in Tafel slope after testing; the post-test Tafel slopes for all IrO_2_ samples were approximately 80 mV/dec, compared to 90–100 mV/dec at beginning of life (Figure S13b, SI). IrO_2_ binds ionomer fragments strongly from −3.7 (Nafion’s SO_3_) to −6.7 eV (Sustainion’s imidazolium), which will lead to blocked or poisoned Ir/O sites. At higher potentials, IrO_2_ can potentially activate degradation mechanisms such as Nafion’s SO_3_ splitting at 1.4 V, Versogen’s double deprotonation at 0.6 V, and Sustainion’s ring oxidation to an alcohol at 1.4 V (Table 1). In addition, Nafion’s SO_3_ can potentially induce dissolution of the IrO_2_ catalyst: isomer IV showcases that SO_3_ can substitute a bridging oxygen, causing it to move to a neighboring surface Ir (see Figure 2). These side reactions may affect OER mechanisms as compared to the stable ionomer-NiO combinations of Versogen/Sustainion-NiO predicted by DFT. Note that, experimentally, current from competing side reactions such as the water formation predicted from theory (Versogen/Sustainion-IrO_2_) would not be distinguishable from OER current in these half-cell measurements.

On IrO_2_ (110), H_2_O spontaneously dissociates into H and OH, thus providing the necessary reactant OH for OER and, therefore, does not inhibit OER performance.^33^ While water formation would not inhibit OER performance, the instability of the ionomer following deprotonation, alcohol oxidation, and fragmental dissociation, i.e. SO_3_ → SO_2_ + O, can affect the availability of active sites and OER activation energies. Since neighboring surface species of O_*x*_H_*y*_ can vary activation energies by up to 0.2 eV, this impact may be more pronounced in the presence of an ionomer, which can influence charge-transfer between key reaction intermediates at the ionomer-catalyst interface.^33^ The activated NiO samples had higher current densities than the IrO_2_ samples, demonstrating that such electrochemical activation may be facilitated by specific anion exchange polymers and be a viable strategy to boosting the performance of non-PGM OER catalysts beyond NiO.

In the case of the catalysts evaluated, IrO_2_ and NiO, electrodes are conditioned prior to oxygen evolution evaluation to ensure an optimum in activity prior to durability testing. In our previous benchmark studies, when not using anion exchange ionomers as a binder (Nafion, polytetrafluoroethylene, no binder), small or minimal changes in catalyst oxygen evolution activity are observed during a 1.8 V hold for 13.5 h.^20,53^ In the case of IrO_2_, a slight decrease is observed due to small amounts of Ir dissolution; in the case of NiO, no activity change is observed due to the minimal dissolution rate of Ni at elevated potential and since the NiO is oxidized (activity losses associated with passivation/oxidation are found when evaluating Ni metal).^20,53^ In both cases (IrO_2_ or NiO), an activity improvement is most likely due to the oxidation of the ionomer, allowing site-access to additional catalyst sites that were previously blocked during catalyst deposition.^20,53^

This improvement may be accentuated in RDE due to the exceptionally thin catalyst layer (limiting delamination, limiting electron transport loss) and the electrolyte (limiting electron and ion transport loss). In a single-cell MEA, however, the ionomer/binder oxidation as described in Table 1 of the functional groups or instability of the hydrocarbon backbone may have more far reaching effects such as catalyst layer delamination, the loss of site access and cell kinetics, increases in Ohmic loss (due to interfacial separation and membrane oxidation at the catalyst layer interface), and increases in catalyst layer resistance (due to the increased tortuosity of electron/ion transport pathways).^54^ However, this joint theoretical-experimental study may explain in part the trends observed here in RDE and in other experimental studies^17,18,31^ of the ionomer’s significant effect on aiding site access, leading to predictive trends of higher performers (NiO-Sustainion, -Versogen), or (in)­stability, resulting in a lower performer (IrO_2_-Versogen) compared to other catalyst-ionomer combinations.

## 3 Conclusion

In this joint theoretical-experimental study of the ionomer-catalyst interface, we provide a comprehensive overview of the degradation mechanisms available to four different classes of ionomers and their possible impact on OER performance and durability: the perfluorinated sulfonic acid Nafion, poly­(aryl piperidinium)-based Versogen, tetramethylimidazolium-formed Sustainion, and the quaternary ammonium-type ETFE, Gen 2, Georgia Tech. Theory elucidated atomic- and electronic-level resolution of the ionomer-catalyst (in)­stability and ionomer-catalyst’s (in)­stability and (in)­activity in the context of the alkaline environment of OH* for anion exchange membrane electrolysis. By examining the potential-dependence of both binding of the ionomer fragment and reactivity of the ionomer to the catalyst and to OH* on the catalyst, theory may explain the difference in activity at break-in as compared to durability testing of half-cell rotating disk electrode experiments. NiO binds the ionomer weakly, allowing for desorption of the ionomer to expose active sites following durability testing: the ionomer may also provide charge-transfer effects to stabilize OH* and favorably influence other steps to OER to enable the considerable improvement of NiO-Versogen and -Sustainion performance by 884 ± 84% and 756 ± 48%, respectively. Improved performance specifically in the catalyst-ionomer combinations for NiO may be due to active site recovery, hydroxide ion shuttling by the ionomer, and enhanced stability of the catalyst. IrO_2_ remains a highly active catalyst for OER but binds the ionomer strongly, limiting available access sites and initiating degradation mechanisms such as SO_3_ splitting, deprotonation, water formation, and alcohol production: electrochemical activity remains comparable at break-in and following durability testing since the ionomer may “stick”, allowing for the same, sustained coverage of O_*x*_H_*y*_ intermediates for OER to occur. Maintained activity by catalyst-ionomer combinations for IrO_2_ may be due to Ir site availability being set during synthesis of catalyst-ionomer inks. This theoretical-experimental study provides insights into the intrinsic properties of multiple classes of ionomers and their interactions with both a platinum group metal catalyst and earth-abundant catalyst, showcasing the chemistry possible not only for AEM electrolysis but also in other applications where these ionomers or catalysts may be utilized such as CO_2_ electrolysis and fuel cells.

## 4 Methods

### 4.1 Theoretical

PW-DFT calculations made use of VASP 5.4.4 ^55–58^ with pseudopotentials generated from the projector augmented wave method^59,60^ and self-consistent break condition of 10^–6^ (10^–5^) eV for the electronic (geometric) loop. Theory employed the Perdew–Burke–Ernzerhof (PBE) functional with Hubbard *U* corrections of *U* = 6.4 for NiO,^61^ this correction allowing for both the correct band-gap for NiO and predicted oxidation energy for the formation enthalpy of NiO + O_2_ → 2NiO_2_, and the solid-state variant of PBE^62^ for IrO_2_, due to its accuracy for reproducing Ir’s 5d splitting in comparison to X-ray photoelectron spectroscopy. The Monkhorst-pack grid of 1 × 1 × 1, shifted in the *x*- and *y*-direction, was utilized for IrO_2_ and 2 × 2 × 1 for NiO. In order to model the ionomer-catalyst interface more realistically, we incorporated implicit solvation (VASPsol)^63–65^ with the dielectric constant chosen to be that of water, in reference to the water-feeds used in experiment. The OH will hydrogen bond to the organic fragments with the bonds elongating to circa 2.0–2.6 Å when solvated with VASPsol (details in Figure 1). In order to consider all the possible geometries of monodentate, bidentate, tridentate, and planar adsorption of these ionomers on the catalyst interface and, additionally, coadsorption of OH* with the ionomer to assess the effect of the alkaline electrolyte and adsorption of the first OER intermediate, this required 1100 calculations per ionomer-catalyst combination, resulting in 8800 calculations ran for the 8 different ionomer-catalyst combinations studied in this paper.

Typically, when calculating the adsorption energies, the reference energies of a single, neutral species e.g. OH radical (less stable species), rather than OH^–^ (more stable species), is utilized since many PW-DFT software packages cannot accurately compute charged species. Notably, software such as jDFTx can accurately compute charged species due to their robust and diverse continuum solvation models but struggle with scaling to the 100+ atoms systems typical of heterogeneous catalysis. The ionomer-IrO_2_ systems in this study often reached >200 atoms. Thus, VASP-based adsorption energies may represent an “over-binding” of the cation or anion species because the energies are referenced to isolated neutral species. The adsorption binding energy was calculated with the equation *E*_ads_ (eV) = *E*_surface+ads_ – *E*_surface_ – *E*_ads_, where the adsorbate is the neutral ionomer or OH. In subsequent coadsorption calculations, where the ionomer-OH complex is utilized as the reference energy for the coadsorbed species on the surface, the adsorption energy is expected to be more accurate and a better representation of the binding strength than the binding strengths utilizing neutral piperidinium and OH. Moreover, the cation-anion complex allows for charge transfer character, where the ionomer can be positive (circa +0.9 e) and the OH negative (circa −0.9 e). Therefore, the coadsorption binding energy was calculated with the equation *E*_ads_ (eV) = *E*_surface+ionomer+OH_ – *E*_surface_ – *E*_ionomer‑OH_. Relative energies and Bader charges of the numerous isomers found are provided in detail in the Supporting Information.

### 4.2 Experimental

All catalyst and ionomer materials were obtained from commercial suppliers and used without further treatment. The OER catalysts evaluated were NiO (US Research Nanomaterials Inc., 99%) and IrO_2_ (Alfa Aesar, 99.99%). The ionomers tested were Nafion perfluorinated resin solution (5 wt % dispersion in water and alcohols, Sigma-Aldrich, 527084), Versogen PiperION Anion Exchange Dispersion (5 wt % dispersion in ethanol, Fuel Cell Store, 72020001), and Sustainion XB-7 Alkaline Ionomer (5 wt % dispersion in ethanol, Dioxide Materials, 68739).

Catalyst inks were formulated to target 100 μg/cm^2^ of metals on polycrystalline Au disc RDE tips (0.196 cm^2^, Pine Research Instrumentation, AFE5T050AU). The inks contained 76 vol % deionized (DI) water (Milli-Q; ⩾18.2 mΩ resistance and <5 ppb organic carbon content) and 24 vol % of *n*-propanol (Sigma-Aldrich, OmniSolv, HPLC Grade). The ionomer content for all polymers was 1 wt % relative to the total solids mass (catalyst plus ionomer). 10 μL portions of the catalyst inks were dropcast on the RDE tips rotating at 100 rotations per minute (RPM), and the rotation speed was increased to 650 (NiO) and 750 (IrO_2_) RPM for drying. The coated RDE tips were then left to dry overnight before use.

All electrochemical tests were performed in 130 mL of 0.1 NaOH electrolyte (Sigma-Aldrich, TraceSELECT grade, 99.9995%) in a custom rotating disc electrode (RDE) Teflon cell with an Au counter electrode and an Hg/HgO reference electrode (Koslow Scientific, 5088 Series). Before testing, the Hg/HgO reference electrode was calibrated vs the reversible hydrogen electrode (RHE) potential. Linear sweep voltammetry was collected at a scan rate of 20 mV/s between 1.2 and 2.0 V vs RHE after five conditioning cycles (1.2 to 1.8 V vs RHE) at 2500 rpm to determine the electrochemical activity of the catalyst. Chronoamperometry at 1.8 V was used as the durability test for all samples. After durability testing, the electrolyte was refreshed, and the reference electrode was recalibrated before assessing the post-test performance. Measured potentials are reported vs the reversible hydrogen reference electrode (RHE) unless otherwise noted. Overpotentials were calculated based on the thermodynamic potential which was adjusted for the nonstandard conditions (nonstandard pressure in Denver of 82.2 kPa) using the Nernst equation (see eq 1 in the SI). All samples were repeated three times for reproducibility.

## Supplementary Material



## Notes

Additional theoretical and experimental details and methodology (PDF)

## Abbreviations

AEM: anion exchange membrane
PEM: proton exchange membrane
PGM: platinum group metal
SO_4_: sulfate
HSO_4_: bisulfate
SO_3_: sulfur trioxide
PW-DFT: plane-wave density functional theory
RDE: rotating disk electrode
OER: oxygen evolution reaction

## References

[1] Pivovar B., Rustagi N., Satyapal S. (2018). Hydrogen at Scale (H 2 @Scale): Key
to a Clean, Economic, and Sustainable Energy System. Electrochem. Soc. Interface.

[2] Ayers K., Danilovic N., Ouimet R., Carmo M., Pivovar B., Bornstein M. (2019). Perspectives
on low-temperature electrolysis and potential
for renewable hydrogen at scale. Annu. Rev.
Chem. Biomol. Eng..

[3] Yun T. G., Sim Y., Lim Y., Kim D., An J.-S., Lee H., Du Y., Chung S.-Y. (2022). Surface
dissolution and amorphization of electrocatalysts
during oxygen evolution reaction: Atomistic features and viewpoints. Mater. Today.

[4] Ghoshal S., Pivovar B. S., Alia S. M. (2021). Evaluating
the effect of membrane-ionomer
combinations and supporting electrolytes on the performance of cobalt
nanoparticle anodes in anion exchange membrane electrolyzers. J. Power Sources.

[5] Li D., Matanovic I., Lee A. S., Park E. J., Fujimoto C., Chung H. T., Kim Y. S. (2019). Phenyl oxidation impacts the durability
of alkaline membrane water electrolyzer. ACS
Appl. Mater. Interfaces.

[6] Kreider M. E., Yu H., Osmieri L., Parimuha M. R., Reeves K. S., Marin D. H., Hannagan R. T., Volk E. K., Jaramillo T. F., Young J. L. (2024). Understanding
the Effects of Anode Catalyst
Conductivity and Loading on Catalyst Layer Utilization and Performance
for Anion Exchange Membrane Water Electrolysis. ACS Catal..

[7] Petrovick J. G., Kushner D. I., Goyal P., Kusoglu A., Radke C. J., Weber A. Z. (2023). Electrochemical measurement of water transport numbers
in anion-exchange membranes. J. Electrochem.
Soc..

[8] Mukerjee S., Yan Y., Xu H. (2021). Hydrogen at scale using
low-temperature anion exchange
membrane electrolyzers. Electrochem. Soc. Interface.

[9] Ito H., Maeda T., Nakano A., Takenaka H. (2011). Properties of Nafion
membranes under PEM water electrolysis conditions. Int. J. Hydrogen Energy.

[10] Schmidt-Rohr K., Chen Q. (2008). Parallel cylindrical
water nanochannels in Nafion fuel-cell membranes. Nature materials.

[11] Gierke, T. ; Hsu, W. The cluster—network model of ion clustering in perfluorosulfonated membranes; ACS Publications: 1982.

[12] Jinnouchi R., Kudo K., Kitano N., Morimoto Y. (2016). Molecular dynamics
simulations on O2 permeation through nafion ionomer on platinum surface. Electrochim. Acta.

[13] Yu T. H., Sha Y., Liu W.-G., Merinov B. V., Shirvanian P., Goddard W. A. (2011). Mechanism for degradation of Nafion
in PEM fuel cells from quantum mechanics calculations. J. Am. Chem. Soc..

[14] Chakraborti T., Sharma R., Krishnamoorthy A. N., Chaudhari H., Mamtani K., Singh J. K. (2024). Unravelling the
effect of molecular
interactions on macroscale properties in Sustainion anion exchange
membrane (AEM) under hydrated conditions using MD simulations. Journal of Membrane Science.

[15] Luo X., Kushner D. I., Kusoglu A. (2023). Anion exchange
membranes: The effect
of reinforcement in water and electrolyte. Journal
of Membrane Science.

[16] Luo X., Rojas-Carbonell S., Yan Y., Kusoglu A. (2020). Structure-transport
relationships of poly (aryl piperidinium) anion-exchange membranes:
Eeffect of anions and hydration. Journal of
Membrane Science.

[17] Krivina R. A., Lindquist G. A., Yang M. C., Cook A. K., Hendon C. H., Motz A. R., Capuano C., Ayers K. E., Hutchison J. E., Boettcher S. W. (2022). Three-Electrode Study of Electrochemical
Ionomer Degradation
Relevant to Anion-Exchange-Membrane Water Electrolyzers. ACS Appl. Mater. Interfaces.

[18] Lindquist G. A., Oener S. Z., Krivina R., Motz A. R., Keane A., Capuano C., Ayers K. E., Boettcher S. W. (2021). Performance
and durability of pure-water-fed anion exchange membrane electrolyzers
using baseline materials and operation. ACS
Appl. Mater. Interfaces.

[19] Volk E. K., Kwon S., Alia S. M. (2023). Catalytic
Activity and Stability
of Non-Platinum Group Metal Oxides for the Oxygen Evolution Reaction
in Anion Exchange Membrane Electrolyzers. J.
Electrochem. Soc..

[20] Anderson G. C., Pivovar B. S., Alia S. M. (2020). Establishing performance
baselines
for the oxygen evolution reaction in alkaline electrolytes. J. Electrochem. Soc..

[21] Ayers K. E., Anderson E. B., Capuano C., Carter B., Dalton L., Hanlon G., Manco J., Niedzwiecki M. (2010). Research advances
towards low cost, high efficiency PEM electrolysis. ECS Trans..

[22] Warren D. S., McQuillan A. J. (2008). Infrared spectroscopic and DFT vibrational mode study
of perfluoro (2-ethoxyethane) sulfonic acid (PES), a model Nafion
side-chain molecule. J. Phys. Chem. B.

[23] Nagao Y. (2013). Highly oriented
sulfonic acid groups in a Nafion thin film on Si substrate. J. Phys. Chem. C.

[24] Curtin D. E., Lousenberg R. D., Henry T. J., Tangeman P. C., Tisack M. E. (2004). Advanced
materials for improved PEMFC performance and life. J. Power Sources.

[25] Mardle P., Chen B., Holdcroft S. (2023). Opportunities
of Ionomer Development
for Anion-Exchange Membrane Water Electrolysis. ACS Energy Letters.

[26] Kutz R. B., Chen Q., Yang H., Sajjad S. D., Liu Z., Masel I. R. (2017). Sustainion imidazolium-functionalized
polymers for
carbon dioxide electrolysis. Energy Technology.

[27] Kohl, P. High-Performance AEM LTE with Advanced Membranes, Ionomers and PGM-Light Electrodes. 2023. https://www.hydrogen.energy.gov/docs/hydrogenprogramlibraries/pdfs/review23/p185_kohl_2023_o-pdf.pdf.

[28] Pivovar, B. Advanced Ionomers & MEAs for Alkaline Membrane Fuel Cells. 2019. https://www.hydrogen.energy.gov/docs/hydrogenprogramlibraries/pdfs/review19/fc147_pivovar_2019_o.pdf.

[29] Wang J., Zhao Y., Setzler B. P., Rojas-Carbonell S., Ben Yehuda C., Amel A., Page M., Wang L., Hu K., Shi L. (2019). Poly­(aryl
piperidinium) membranes and ionomers
for hydroxide exchange membrane fuel cells. Nat. Energy.

[30] Ha M.-A., Alia S. M., Norman A. G., Miller E. M. (2024). Fe-Doped
Ni-Based
Catalysts Surpass Ir-Baselines for Oxygen Evolution Due to Optimal
Charge-Transfer Characteristics. ACS Catal..

[31] Volk E., Kwon S., Alia S. M. (2023). Catalytic
Activity and Stability
of Non-Platinum Group Metal Oxides for the Oxygen Evolution Reaction
in Anion Exchange Membrane Electrolyzers. J.
Electrochem. Soc..

[32] Alia S. M., Reeves K. S., Yu H., Park J., Kariuki N., Kropf A. J., Myers D. J., Cullen D. A. (2022). Electrolyzer Performance
Loss from Accelerated Stress Tests and Corresponding Changes to Catalyst
Layers and Interfaces. J. Electrochem. Soc..

[33] Ha M.-A., Larsen R. E. (2021). Multiple
Reaction Pathways for the Oxygen Evolution
Reaction May Contribute to IrO2 (110)’s High Activity. J. Electrochem. Soc..

[34] Marković N. M., Adžić R. R., Cahan B. D., Yeager E. B. (1994). Structural
effects in electrocatalysis: oxygen reduction on platinum low index
single-crystal surfaces in perchloric acid solutions. J. Electroanal. Chem..

[35] Markovic N. M., Gasteiger H. A., Ross P. N. (1995). Oxygen reduction
on platinum low-index single-crystal surfaces in sulfuric acid solution:
rotating ring-Pt (hkl) disk studies. J. Phys.
Chem..

[36] Danilovic N., Subbaraman R., Chang K.-C., Chang S. H., Kang Y. J., Snyder J., Paulikas A. P., Strmcnik D., Kim Y.-T., Myers D. (2014). Activity–stability trends for the oxygen evolution
reaction on monometallic oxides in acidic environments. J. Phys. Chem. Lett..

[37] Motealleh B., Liu Z., Masel R. I., Sculley J. P., Ni Z. R., Meroueh L. (2021). Next-generation
anion exchange membrane water electrolyzers operating for commercially
relevant lifetimes. Int. J. Hydrogen Energy.

[38] Liu Z., Sajjad S. D., Gao Y., Kaczur J., Masel R. (2017). An alkaline
water electrolyzer with sustainion membranes: 1 a/cm^2^ at
1.9 v with base metal catalysts. ECS Trans..

[39] Liu Z., Sajjad S. D., Gao Y., Yang H., Kaczur J. J., Masel R. I. (2017). The effect of membrane
on an alkaline water electrolyzer. Int. J. Hydrogen
Energy.

[40] Sediva E., Bonizzoni S., Caielli T., Mustarelli P. (2023). Distribution
of relaxation times as an accessible method to optimize the electrode
structure of anion exchange membrane fuel cells. J. Power Sources.

[41] Goncalves
Biancolli A. L., Herranz D., Wang L., Stehlikova G., Bance-Soualhi R., Ponce-Gonzalez J., Ocon P., Ticianelli E. A., Whelligan D. K., Varcoe J. R., Santiago E. I. (2018). ETFE-based anion-exchange
membrane ionomer powders for alkaline membrane fuel cells: a first
performance comparison of head-group chemistry. J. Mater. Chem. A.

[42] Volk E. K., Clauser A. L., Kreider M. E., Soetrisno D. D., Khandavalli S., Sugar J. D., Kwon S., Alia S. M. (2025). Role of
the Ionomer in Supporting Electrolyte-Fed Anion Exchange Membrane
Water Electrolyzers. ACS Electrochemistry.

[43] Alia S. M., Anderson G. C. (2019). Iridium Oxygen Evolution
Activity and Durability Baselines
in Rotating Disk Electrode Half-Cells. J. Electrochem.
Soc..

[44] Alia S. M., Ha M.-A., Anderson G. C., Ngo C., Pylypenko S., Larsen R. E. (2019). The roles of oxide growth and sub-surface facets in
oxygen evolution activity of iridium and its impact on electrolysis. J. Electrochem. Soc..

[45] Alia S. M., Manco J., Anderson G. C., Hurst K. E., Capuano C. B. (2021). The Effect
of Material Properties on Oxygen Evolution Activity and Assessing
Half-Cell Screening as a Predictive Tool in Electrolysis. J. Electrochem. Soc..

[46] Alia S. M., Rasimick B., Ngo C., Neyerlin K., Kocha S. S., Pylypenko S., Xu H., Pivovar B. S. (2016). Activity and durability
of iridium nanoparticles in the oxygen evolution reaction. J. Electrochem. Soc..

[47] Alia S. M., Reeves K. S., Cullen D. A., Yu H., Kropf A. J., Kariuki N., Park J. H., Myers D. J. (2024). Simulated
start-stop
and the impact of catalyst layer redox on degradation and performance
loss in low-temperature electrolysis. J. Electrochem.
Soc..

[48] Alia S. M., Reeves K. S., Baxter J. S., Cullen D. A. (2020). The impact
of ink
and spray variables on catalyst layer properties, electrolyzer performance,
and electrolyzer durability. J. Electrochem.
Soc..

[49] Bockris J. M. (1956). Kinetics
of activation controlled consecutive electrochemical reactions: anodic
evolution of oxygen. J. Chem. Phys..

[50] Trotochaud L., Young S. L., Ranney J. K., Boettcher S. W. (2014). Nickel–iron
oxyhydroxide oxygen-evolution electrocatalysts: the role of intentional
and incidental iron incorporation. J. Am. Chem.
Soc..

[51] Bode H., Dehmelt K., Witte J. (1966). Zur kenntnis der nickelhydroxidelektrode—I.Über
das nickel (II)-hydroxidhydrat. Electrochim.
Acta.

[52] Louie M. W., Bell A. T. (2013). An Investigation of Thin-Film Ni–Fe
Oxide Catalysts
for the Electrochemical Evolution of Oxygen. J. Am. Chem. Soc..

[53] Alia S. M., Pivovar B. S. (2018). Evaluating Hydrogen
Evolution and Oxidation in Alkaline
Media to Establish Baselines. J. Electrochem.
Soc..

[54] Alia, S. M. HydroGEN: Low Temperature Electrolysis; Department of Energy. U.S. Department of Energy, 2023. https://www.hydrogen.energy.gov/docs/hydrogenprogramlibraries/pdfs/review23/p148a_alia_2023_p-pdf.pdf (accessed October 1, 2024).

[55] Kresse G., Hafner J. (1993). Ab initio molecular dynamics for liquid metals. Phys. Rev. B.

[56] Kresse G., Hafner J. (1994). Ab initio molecular-dynamics simulation of the liquid-metal–amorphous-semiconductor
transition in germanium. Phys. Rev. B.

[57] Kresse G., Furthmüller J. (1996). Efficiency
of ab-initio total energy calculations for
metals and semiconductors using a plane-wave basis set. Comput. Mater. Sci..

[58] Kresse G., Furthmüller J. (1996). Efficient
iterative schemes for ab initio total-energy
calculations using a plane-wave basis set. Phys.
Rev. B.

[59] Blöchl P. E. (1994). Projector
augmented-wave method. Phys. Rev. B.

[60] Kresse G., Joubert D. (1999). From ultrasoft pseudopotentials
to the projector augmented-wave
method. Phys. Rev. B.

[61] Wang L., Maxisch T., Ceder G. (2006). Oxidation
energies of transition
metal oxides within the GGA+ U framework. Phys.
Rev. B.

[62] Perdew J. P., Ruzsinszky A., Csonka G. I., Vydrov O. A., Scuseria G. E., Constantin L. A., Zhou X., Burke K. (2008). Restoring
the density-gradient
expansion for exchange in solids and surfaces. Phys. Rev. Lett..

[63] Mathew K., Kolluru V., Mula S., Steinmann S. N., Hennig R. G. (2019). Implicit self-consistent electrolyte
model in plane-wave
density-functional theory. J. Chem. Phys..

[64] Mathew, K. ; Hennig, R. G. Implicit self-consistent description of electrolyte in plane-wave density-functional theory. arXiv preprint arXiv:1601.03346, 2016. 10.1063/1.5132354 31864239

[65] Mathew K., Sundararaman R., Letchworth-Weaver K., Arias T., Hennig R. G. (2014). Implicit
solvation model for density-functional study of nanocrystal surfaces
and reaction pathways. J. Chem. Phys..

