# Supplementary material for: Complex Degradation Mechanisms Accessible to Anion Exchange Membrane Ionomers on Model Catalysts, NiO and IrO2
Source: ACS Electrochem. 2025 Apr 29;1(8):1339–51. doi: 10.1021/acselectrochem.5c00040 (PMC12337097; doi:10.1021/acselectrochem.5c00040)
Supplement: Supplementary file 1 [file ec5c00040_si_001.pdf]

Supporting Information

Complex Degradation Mechanisms Accessible to Anion Exchange Membrane Ionomers on Model Catalysts, NiO and IrO_2

Mai-Anh Ha[1,,z] Emily K. Volk,[2,3] Oliver Leitner,[1] Avital Isakov,[1] Héctor J. González Vélez,[4] Shaun Alia,[2] Ross Larsen[1]*

[1] Computational Science Center, National Renewable Energy Laboratory, 15013 Denver West Parkway, Golden, Colorado 80401, United States,

[2] Chemistry and Nanoscience Center, National Renewable Energy Laboratory, 15013 Denver West Parkway, Golden, Colorado 80401, United States,

[3] Advanced Energy Systems Graduate Program, Colorado School of Mines, Golden Colorado 80401, United States

[4] Department of Chemistry, University of Puerto Rico Humacao Campus, Humacao 00792, Puerto Rico

Corresponding Author

[*] MaiAnh.Ha@nrel.gov

Table of Contents

Theoretical SI Figures and Tables **S2-S19**

Figure S1. SO_3 and SO_3-OH isomers with Bader charges, bond distances, and total energies. **S2**

Figure S2. SO_3 and SO_3-OH isomers on NiO (100) with Bader charges, bond distances, and total energies. **S2-S3**

Table S1. Bader Charges of Isomer V-a through V-l for SO_3-OH isomers on NiO (100) **S4**

Figure S3. SO_3 and SO_3-OH isomers on IrO_2 (110) with Bader charges and relative energies. **S4-S5**

Figure S4. 4methyls-methyl imidazolium with Bader charges, bond distances, and total energies. **S5**

Figure S5. 4methyls-methyl imidazolium and 4methyls-methyl imidazolium-OH isomers on NiO (100) with Bader charges and relative energies. **S6-S8**

Table S2. Bader Charges of Isomer I-a through I-l **S9**

Figure S6. 4methyls-methyl imidazolium and 4methyls-methyl imidazolium-OH isomers on IrO_2 (110) with Bader charges and relative energies. **S9-S10**

Figure S7. Piperidinium with Bader charges, bond distances, and total energies. **S10**

Figure S8. Piperidinium and Piperidinium-OH isomers isomers on NiO (100) with Bader charges and relative energies. **S10-S13**

Figure S9. Piperidinium and Piperidinium-OH isomers on IrO_2 (110) with Bader charges and relative energies. **S14-S16**

Figure S10. $N(CH_3)_4$ and $N(CH_3)_4$-OH isomers with Bader charges, bond distances, and total energies. **S17**

Figure S11. $N(CH_3)_4$ and $N(CH_3)_4$-OH isomers on NiO (100) with Bader charges and relative energies. **S17-S18**

Figure S12. $N(CH_3)_4$ and $N(CH_3)_4$-OH isomers on IrO_2 (110) with Bader charges and relative energies. **S19**

Experimental SI Figures and Tables **S20**

Figure S13. Tafel slopes before and after testing for a) NiO and b) IrO_2 with Nafion, Versogen, and Sustainion ionomers.

References **S20**

Theoretical

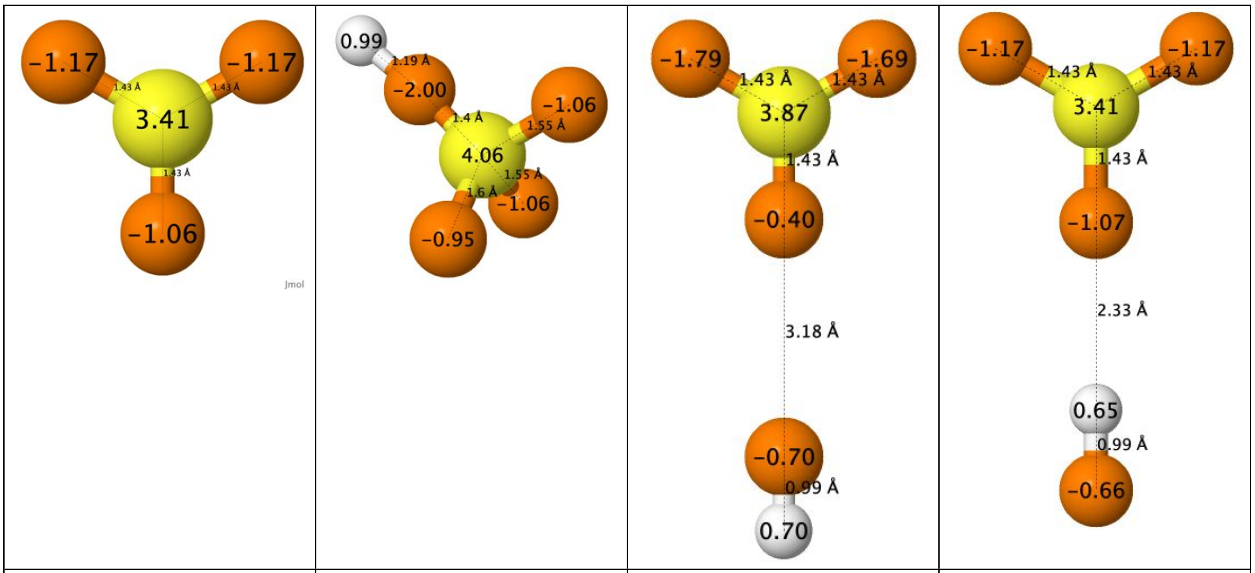

Iso I, SO_3	Iso I, HSO_4	Iso II, SO_3-OH	Iso III, SO_3-OH
E_{tot} (eV) = -23.197	E_{tot} (eV) = -44.253	E_{tot} (eV) = -31.140	E_{tot} (eV) = -31.120

Figure S1. SO_3 and SO_3-OH isomers with Bader charges, bond distances, and total energies.

a) SO_3 on NiO

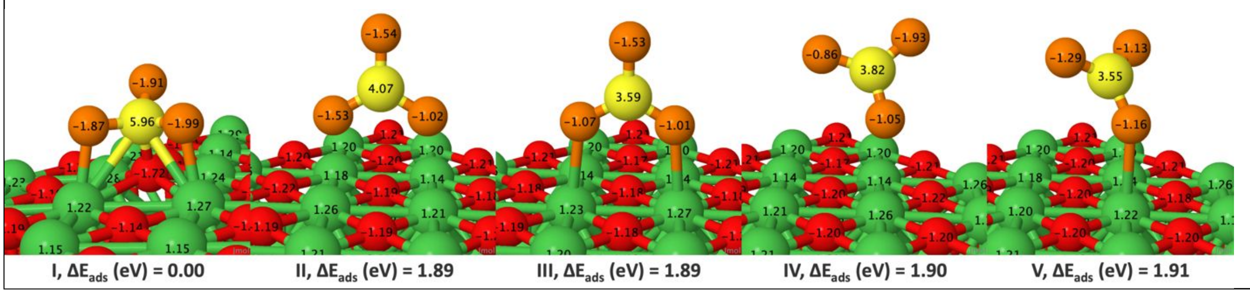

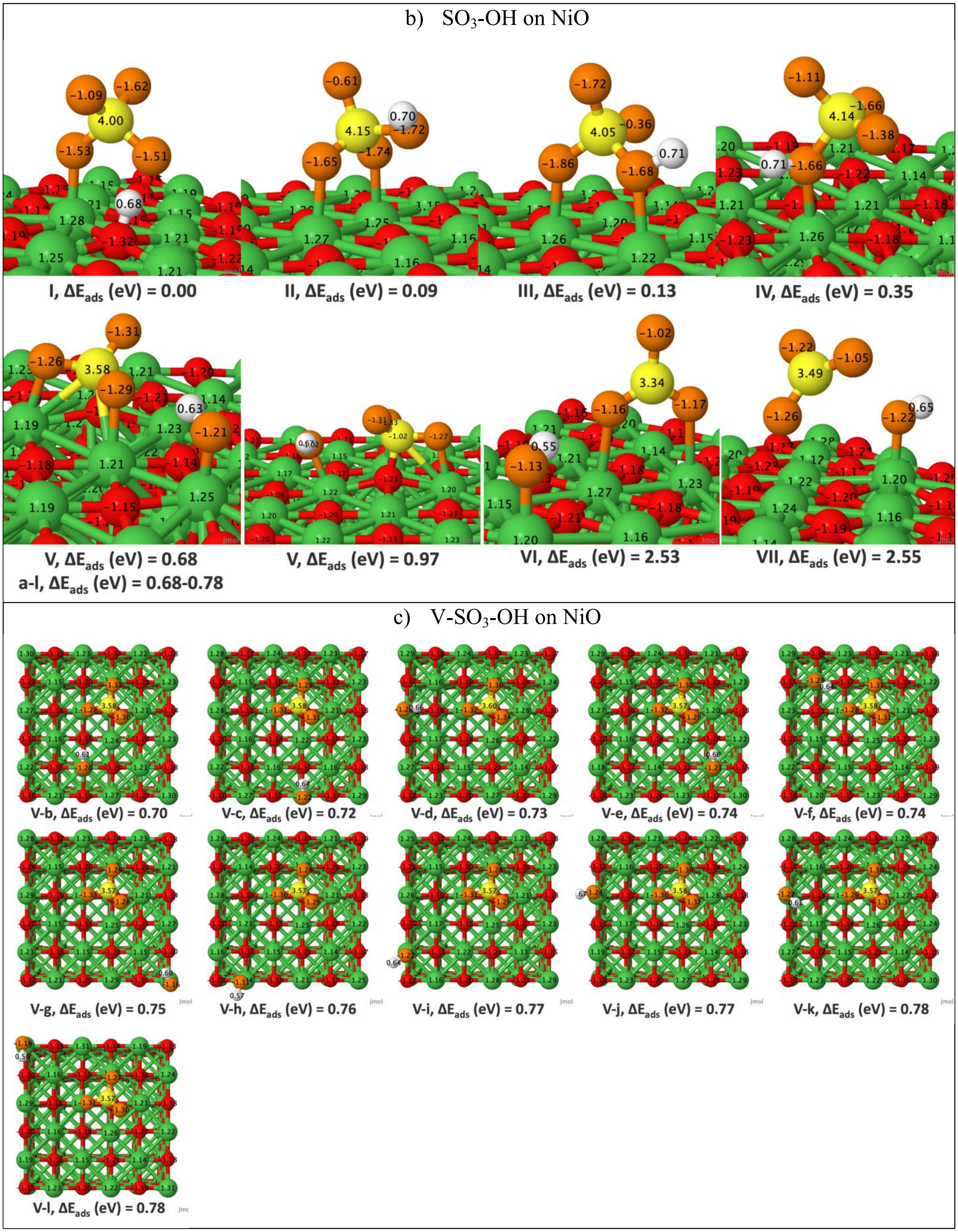

Figure S2. SO_3 and SO_3-OH isomers on NiO (100) with Bader charges, bond distances, and total energies.

Table S1. Bader Charges of Isomer V-a through V-l for SO_3-OH isomers on NiO (100)

Isomer V	ΔQ_{SO3} (e)	ΔQ_{OH} (e)
a	-0.28	-0.57
b	-0.32	-0.59
c	-0.33	-0.59
d	-0.37	-0.61
e	-0.34	-0.61
f	-0.32	-0.59
g	-0.33	-0.56
h	-0.31	-0.54
i	-0.31	-0.58
j	-0.34	-0.57
k	-0.33	-0.61
l	-0.30	-0.62

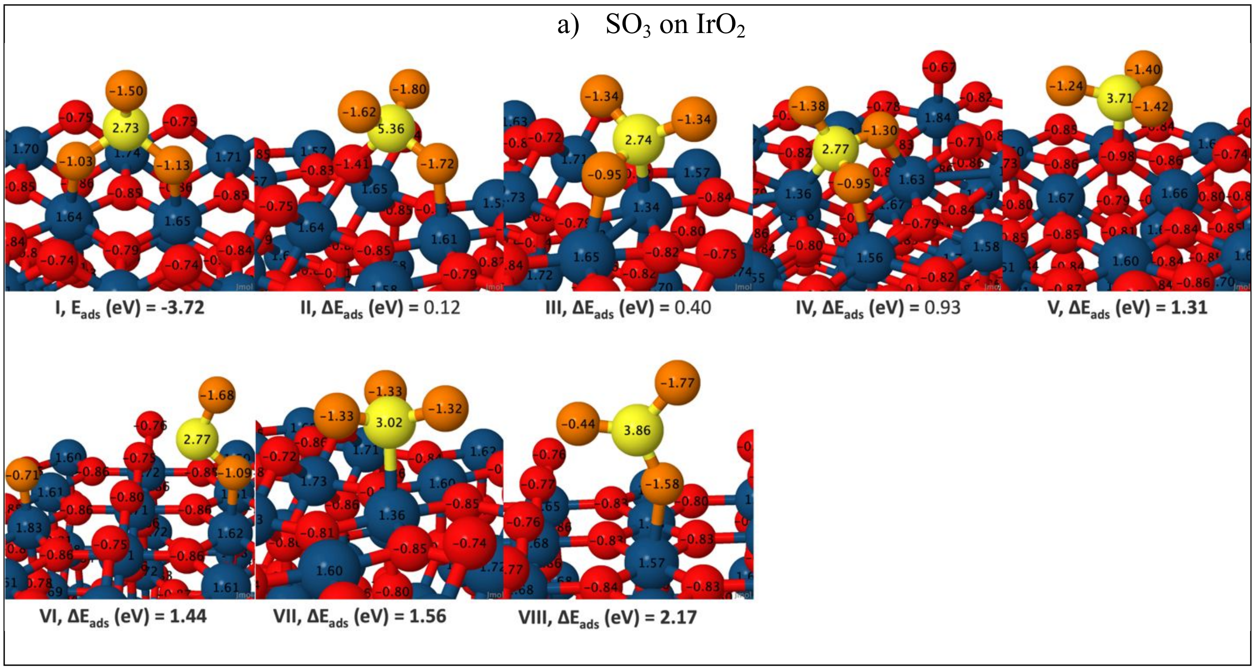

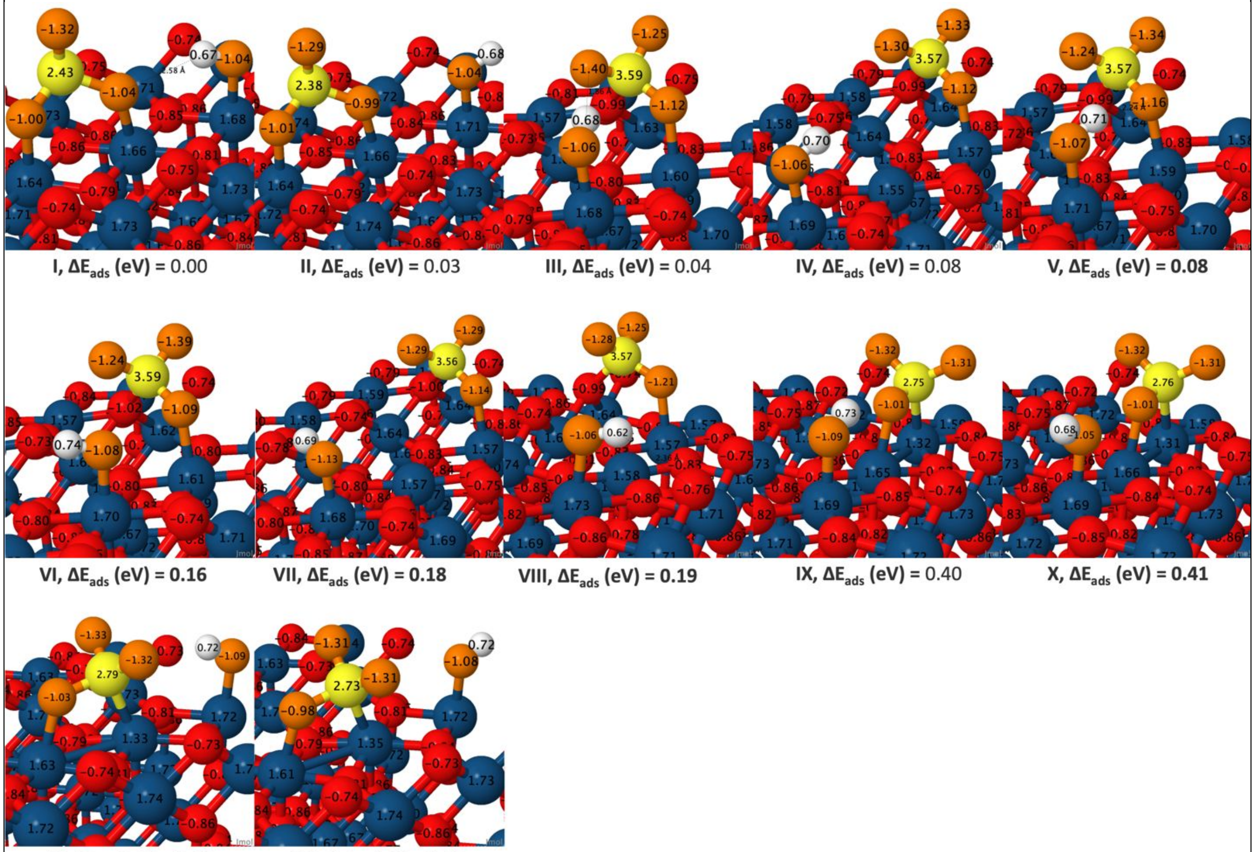

Figure S3. SO_3 and SO_3-OH isomers on IrO_2 (110) with Bader charges and relative energies.

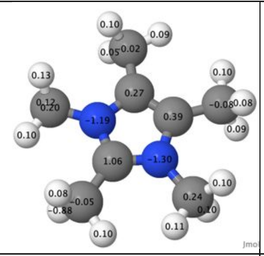	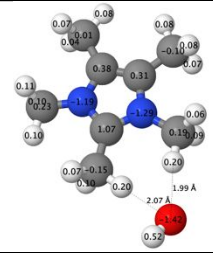	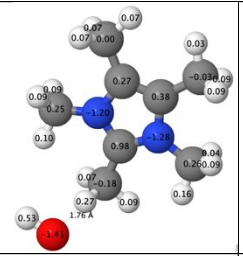	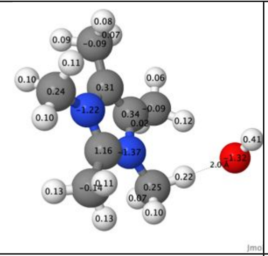	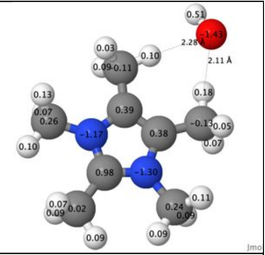
**Iso I, 4methyls-methyl-imidazolium** **E_{tot} (eV) = -142.478**	**Iso I, 4methyls-methyl-imidazolium—OH** **E_{tot} (eV) = -154.317** **Δ**Q (e) = ±0.90	Iso II, 4methyls-methyl-imidazolium—OH E_{tot} (eV) = -154.289 **Δ**Q (e) = ±0.88	Iso III, 4methyls-methyl-imidazolium—OH E_{tot} (eV) = -154.270 **Δ**Q (e) = ±0.91	Iso IV, 4methyls-methyl-imidazolium—OH E_{tot} (eV) = -154.230 **Δ**Q (e) = ±0.92

Figure S4. 4methyls-methyl imidazolium with Bader charges, bond distances, and total energies.

a) Sustainion on NiO

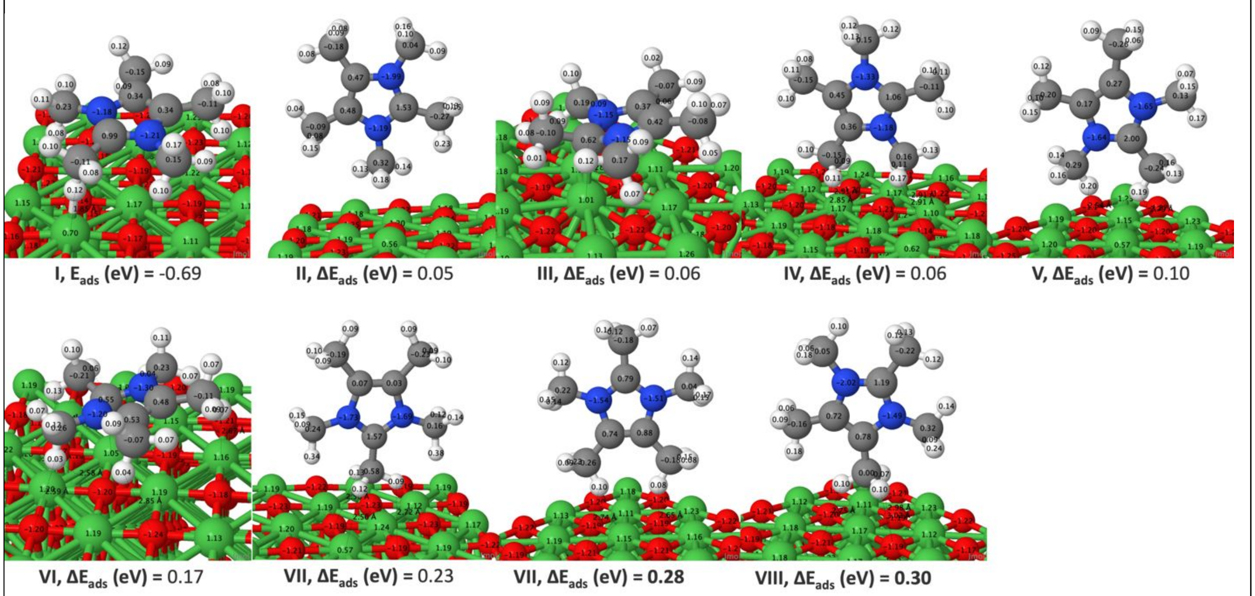

b) Sustainion-OH on NiO

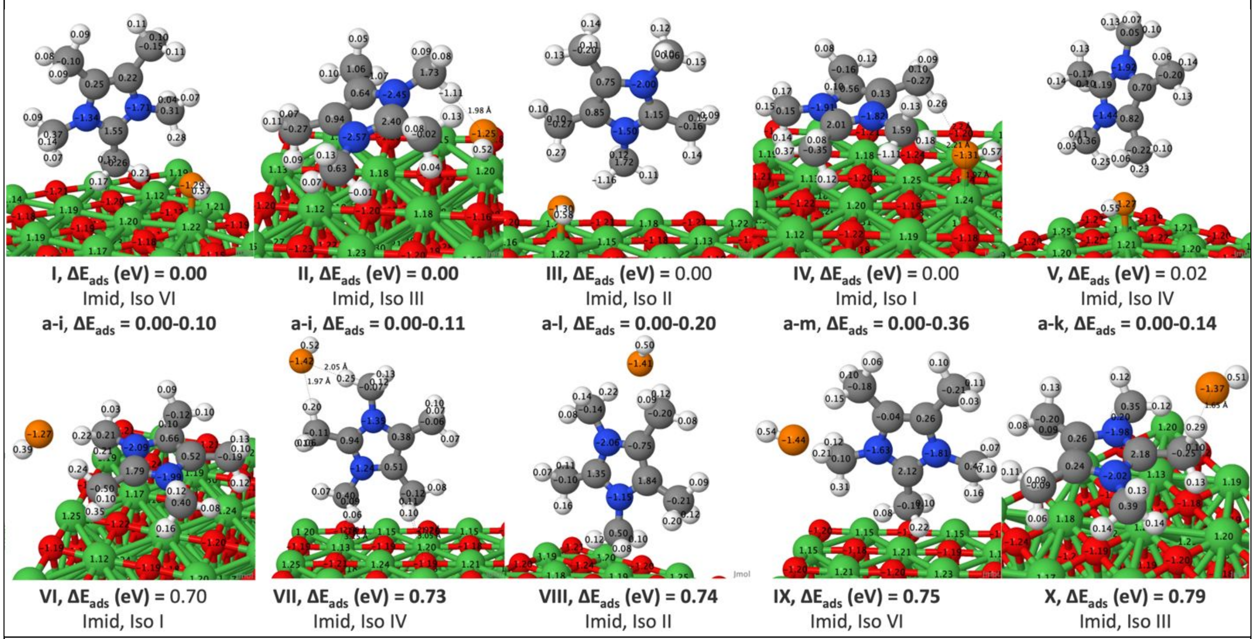

c) I- Sustainion-OH on NiO

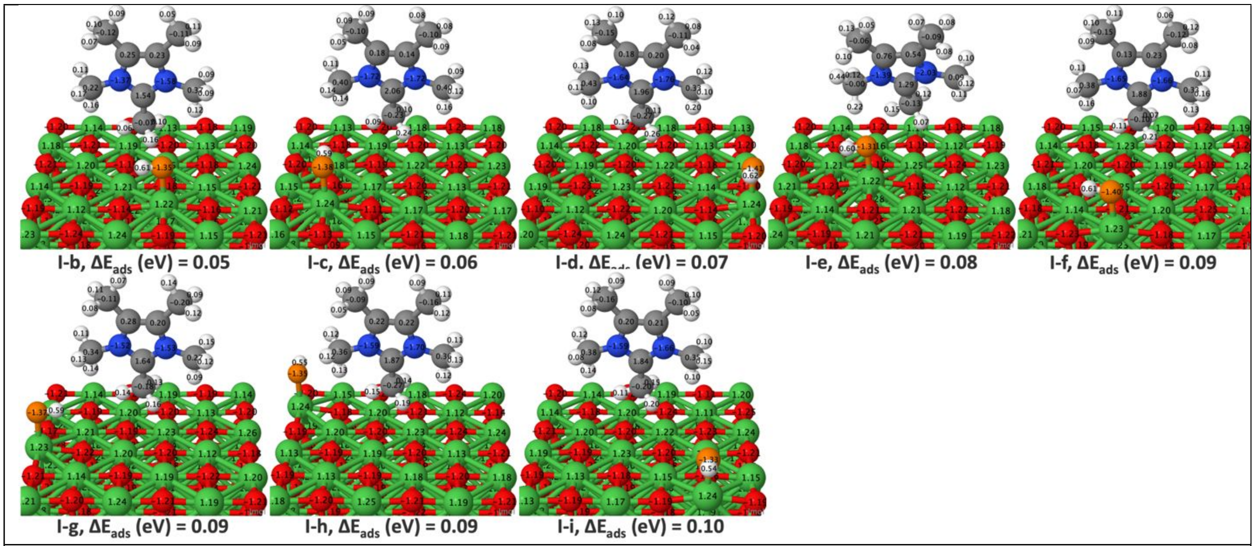

d) II- Sustainion-OH on NiO

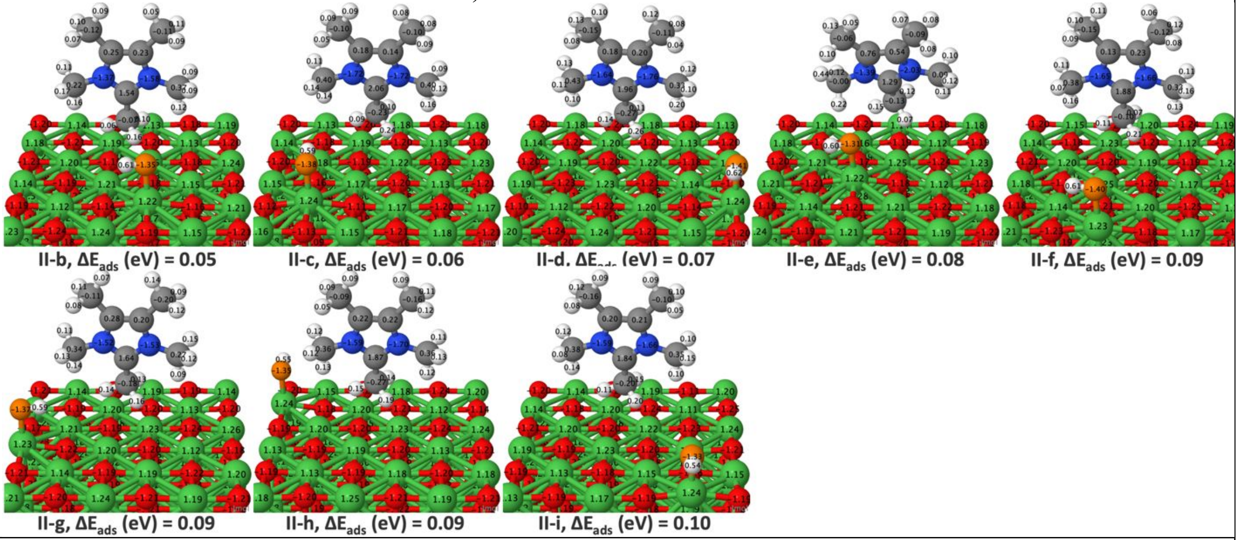

e) III- Sustainion-OH on NiO

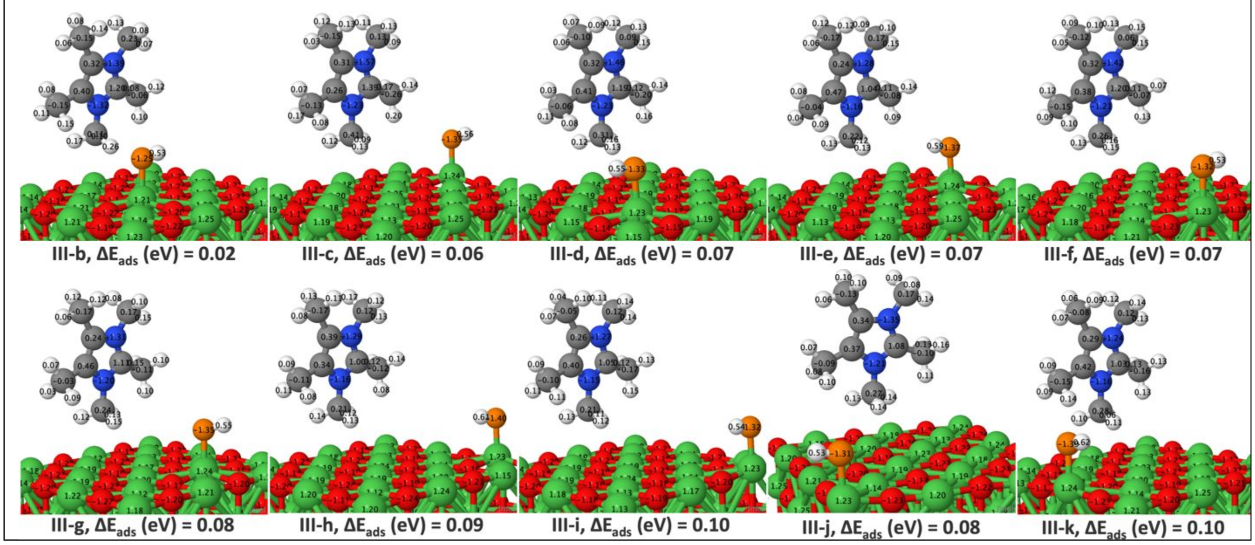

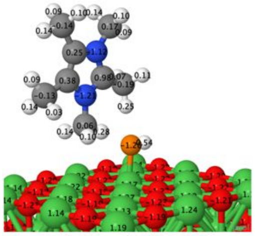

III-l, ΔE_{ads} (eV) = 0.20

f) IV-Sustainion-OH on NiO

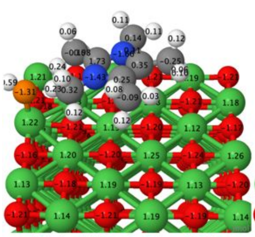

IV-b, ΔE_{ads} (eV) = 0.02

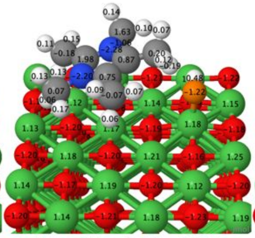

IV-c, ΔE_{ads} (eV) = 0.04

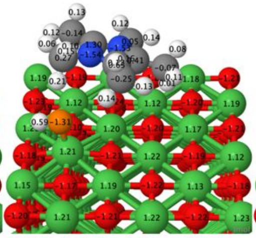

IV-d, ΔE_{ads} (eV) = 0.06

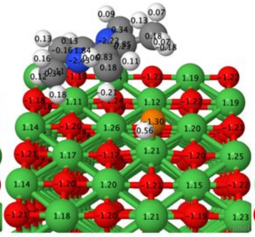

IV-e, ΔE_{ads} (eV) = 0.08

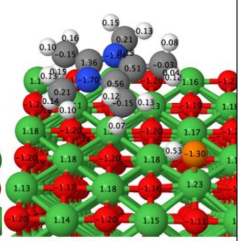

IV-f, ΔE_{ads} (eV) = 0.08

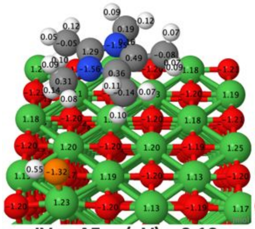

IV-g, ΔE_{ads} (eV) = 0.10

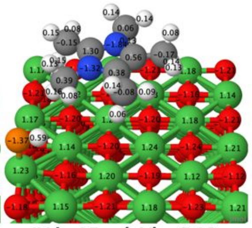

IV-h, ΔE_{ads} (eV) = 0.10

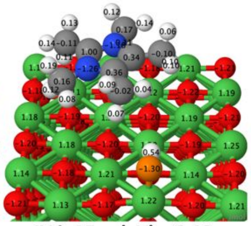

IV-i, ΔE_{ads} (eV) = 0.13

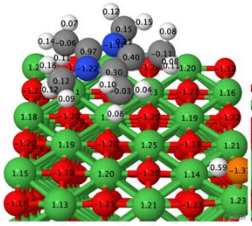

IV-j, ΔE_{ads} (eV) = 0.14

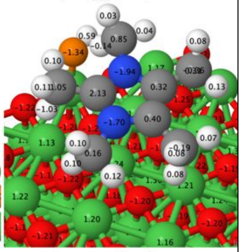

IV-k, ΔE_{ads} (eV) = 0.14

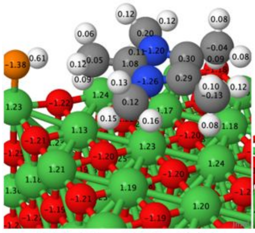

IV-l, ΔE_{ads} (eV) = 0.15

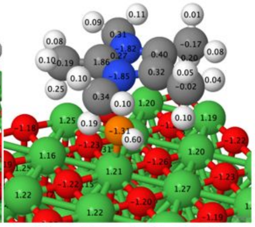

IV-m, ΔE_{ads} (eV) = 0.36

g) V-Sustainion-OH on NiO

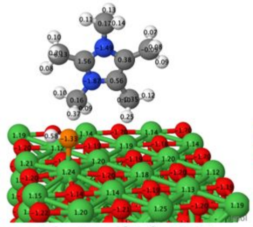

V-b, ΔE_{ads} (eV) = 0.03

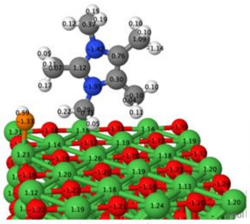

V-c, ΔE_{ads} (eV) = 0.04

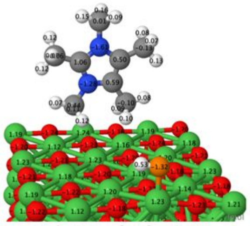

V-d, ΔE_{ads} (eV) = 0.09

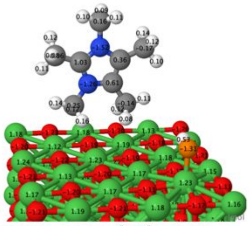

V-e, ΔE_{ads} (eV) = 0.10

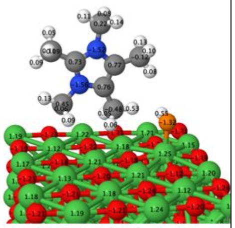

V-f, ΔE_{ads} (eV) = 0.10

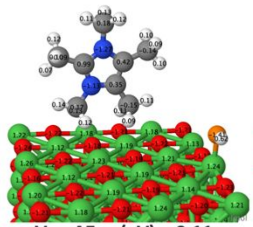

V-g, ΔE_{ads} (eV) = 0.11

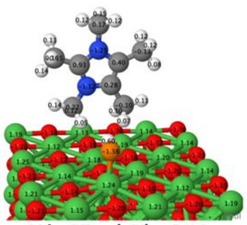

V-h, ΔE_{ads} (eV) = 0.11

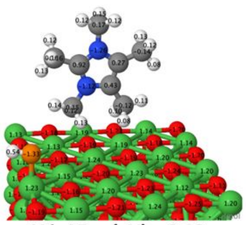

V-i, ΔE_{ads} (eV) = 0.13

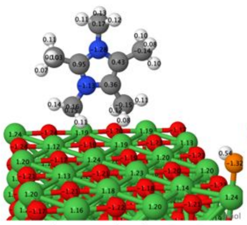

V-j, ΔE_{ads} (eV) = 0.13

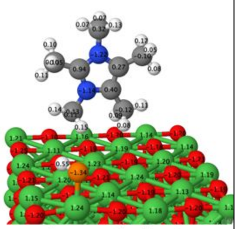

V-k, ΔE_{ads} (eV) = 0.14

Figure S5. 4methyls-methyl imidazolium and 4methyls-methyl imidazolium-OH isomers on NiO (100) with Bader charges and relative energies.

Table S2. Bader Charges of Isomer I-a through I-l

Isomer I	**Δ**Q_{SO3} (e)	**Δ**Q_{OH} (e)
a	-0.28	-0.57
b	-0.32	-0.59
c	-0.33	-0.59
d	-0.37	-0.61
e	-0.34	-0.61
f	-0.32	-0.59
g	-0.33	-0.56
h	-0.31	-0.54
i	-0.31	-0.58

a) Sustainion on IrO_2

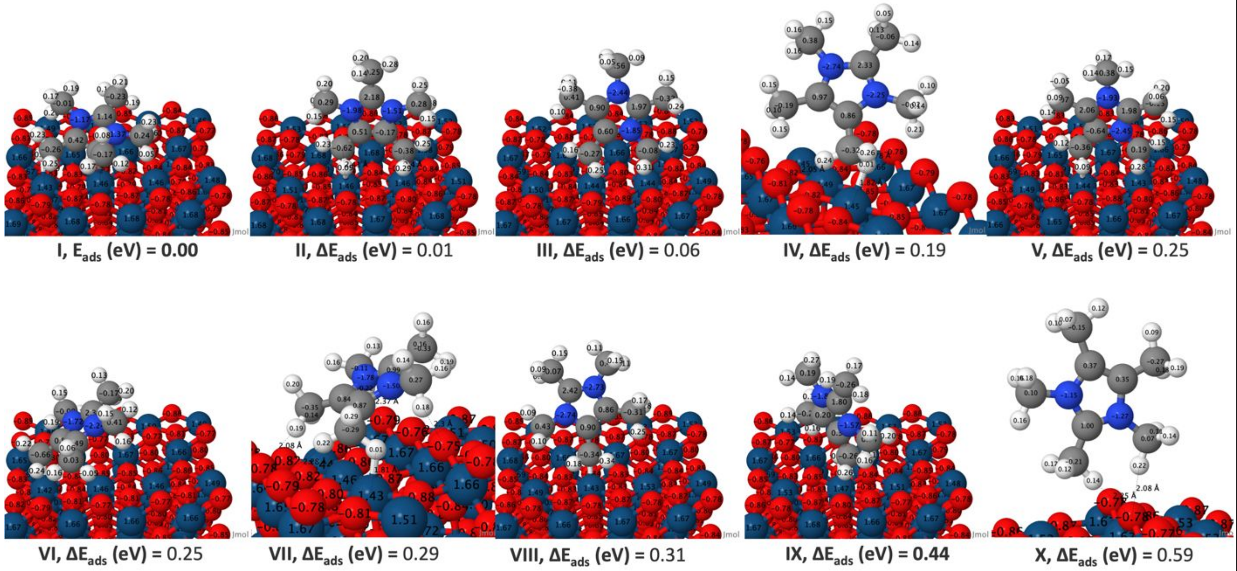

b) Sustainion-OH on IrO_2

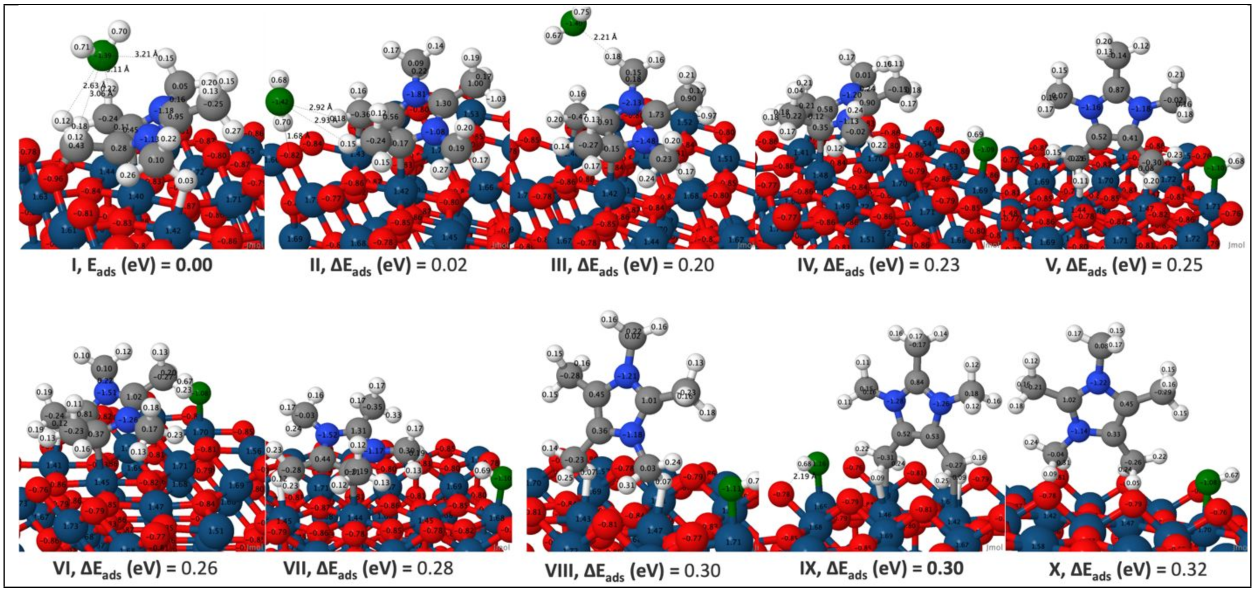

Figure S6. 4methyls-methyl imidazolium and 4methyls-methyl imidazolium-OH isomers on IrO_2 (110) with Bader charges and relative energies.

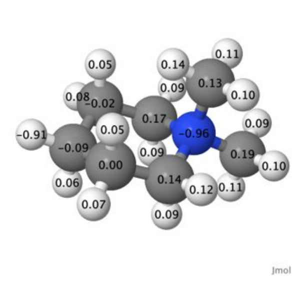	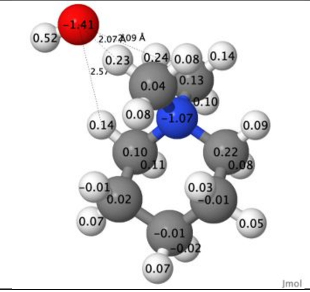	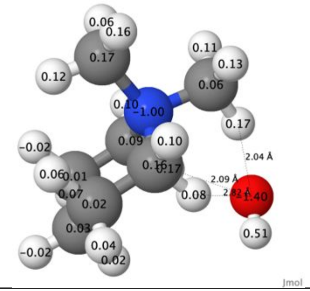	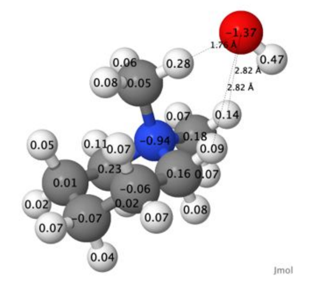
**Iso I, Piperidinium**<br>**E_{tot} (eV) = -128.289**	**Iso I, Piperidinium — OH**<br>**E_{tot} (eV) = -140.122**	Iso II, Piperidinium — OH<br>E_{tot} (eV) = -140.113	Iso III, Piperidinium— OH<br>E_{tot} (eV) = -140.077
	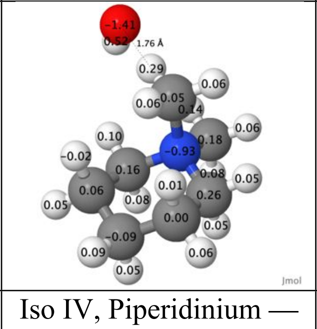	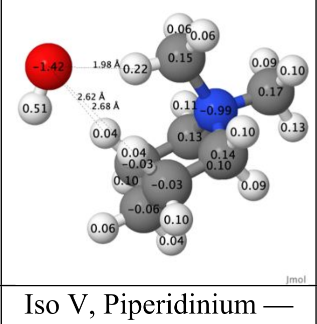	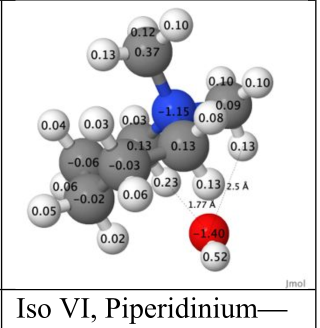
	Iso IV, Piperidinium — OH<br>E_{tot} (eV) = -140.075	Iso V, Piperidinium — OH<br>E_{tot} (eV) = -140.070	Iso VI, Piperidinium— OH<br>E_{tot} (eV) = -140.064

Figure S7. Piperidinium with Bader charges, bond distances, and total energies.

a) Piperidinium on NiO

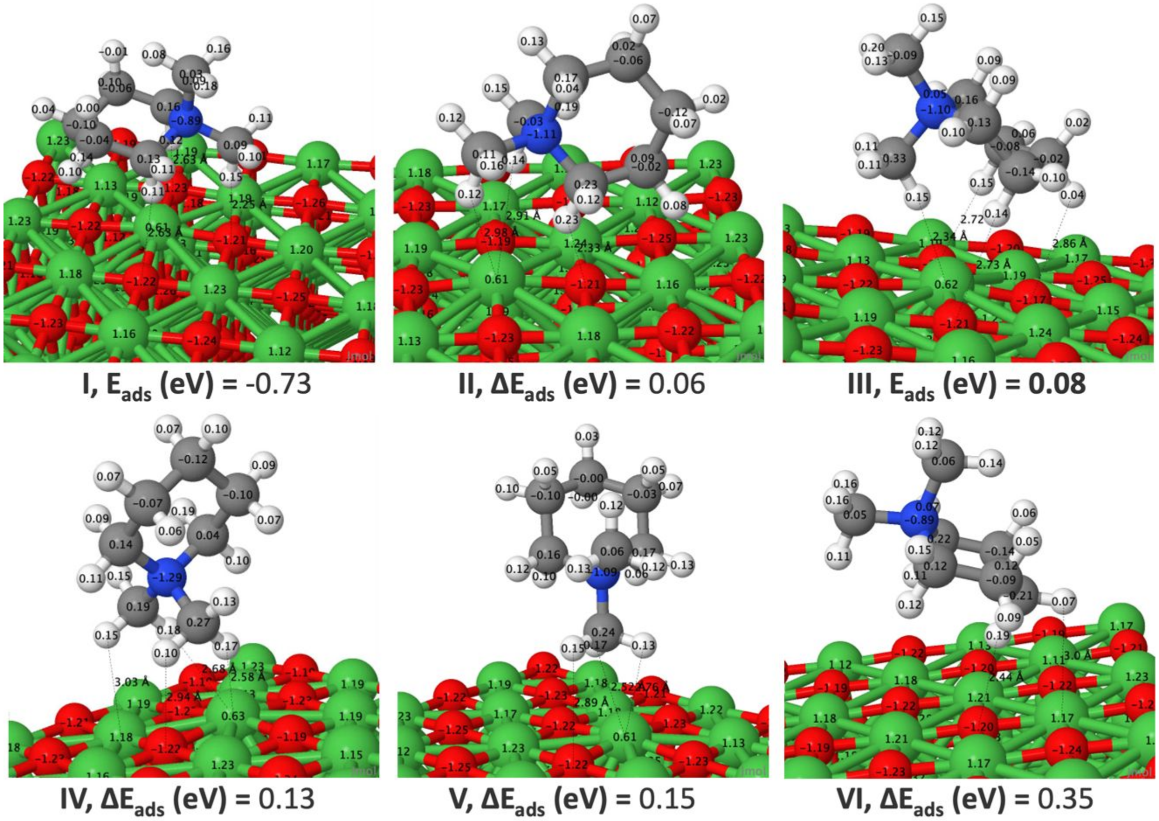

b) Piperidinium-OH on NiO

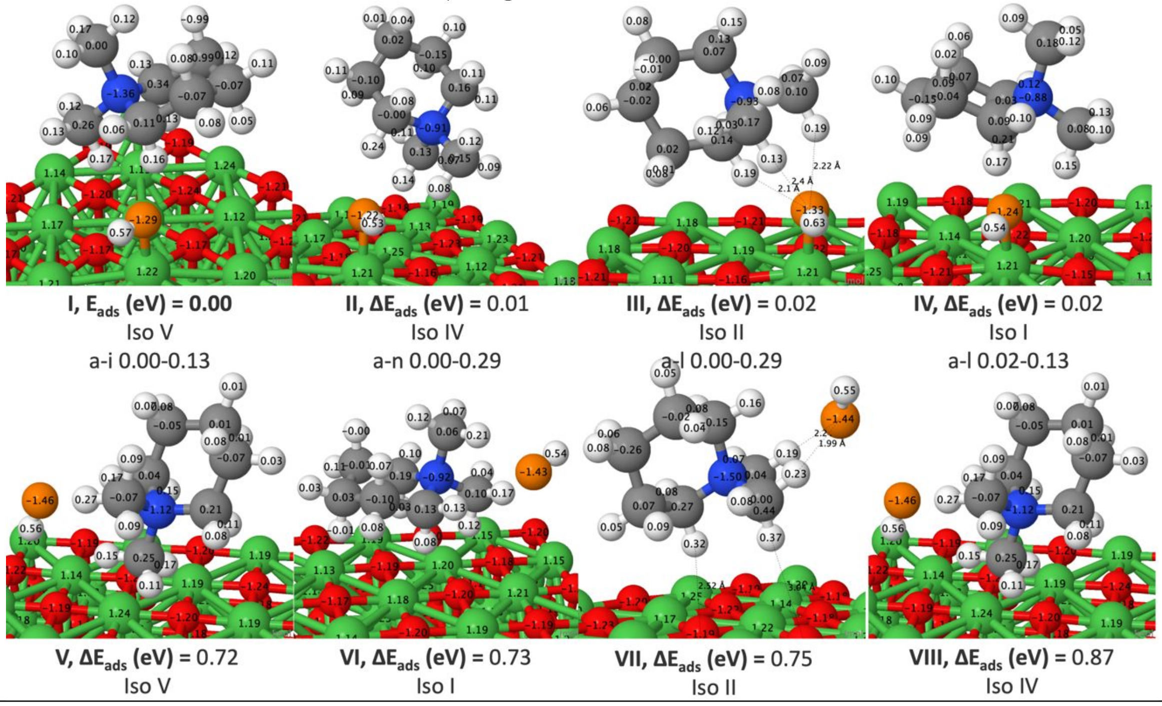

c) I-Piperidinium-OH on NiO

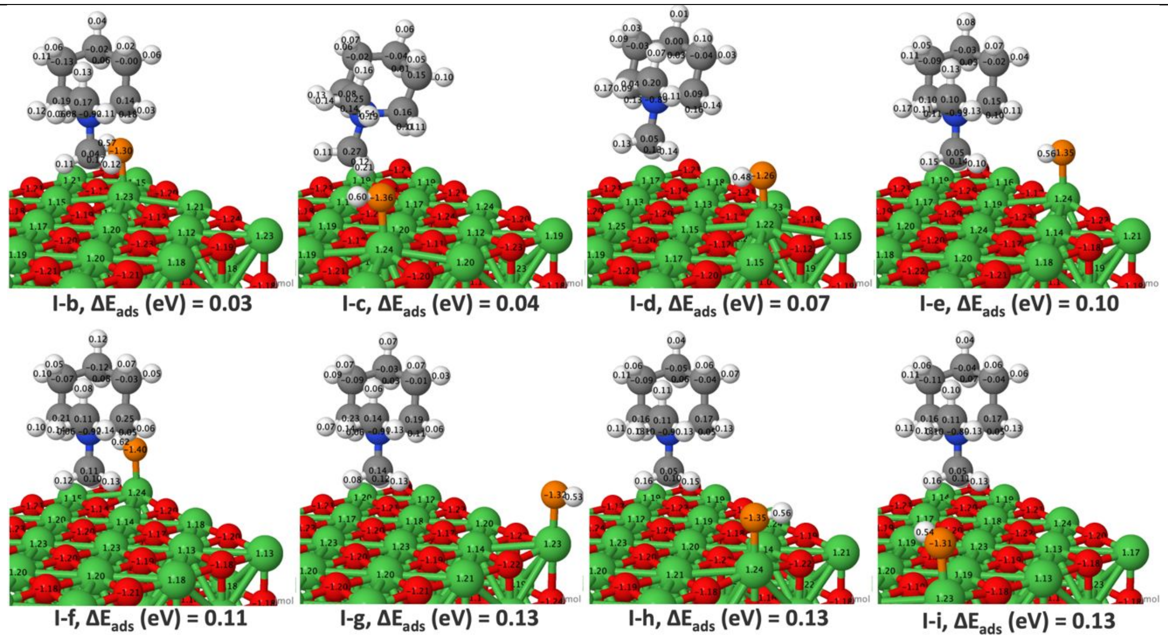

d) II- Piperidinium-OH on NiO

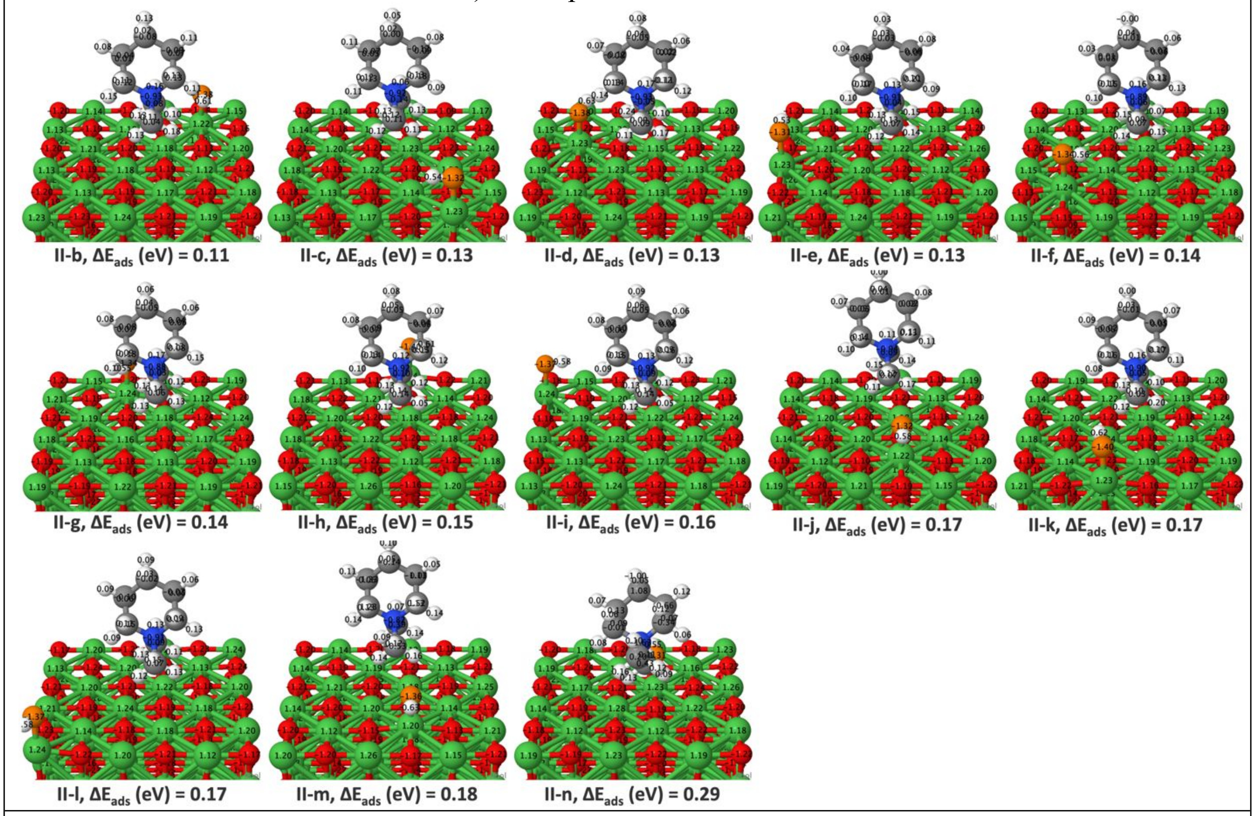

e) III- Piperidinium-OH on NiO

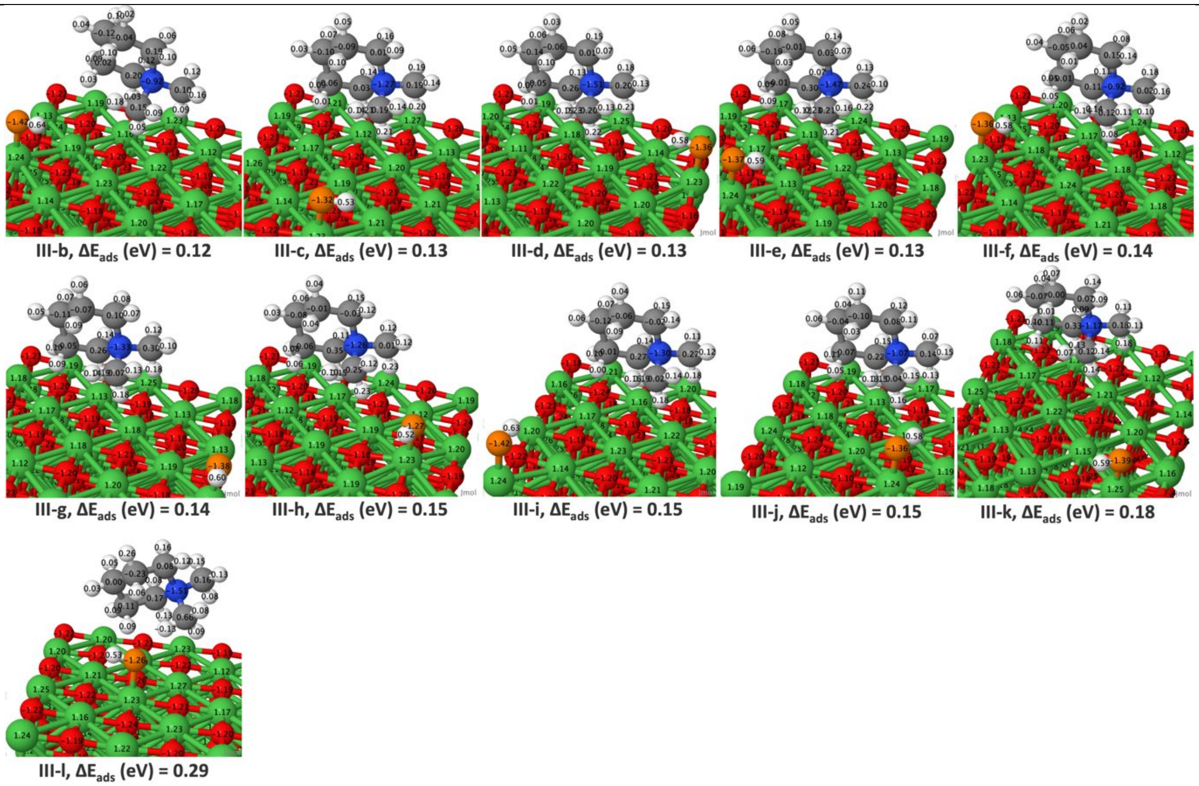

f) IV- Piperidinium-OH on NiO

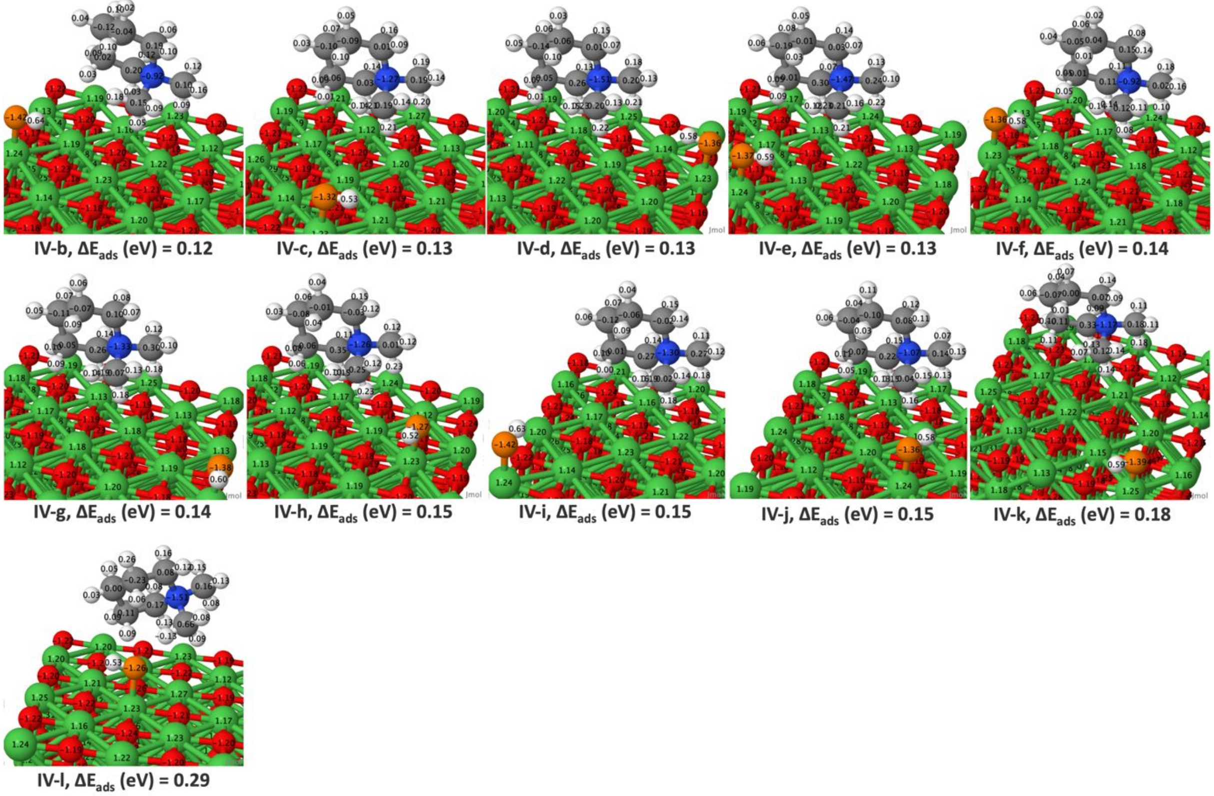

Figure S8. Piperidinium and Piperidinium-OH isomers isomers on NiO (100) with Bader charges and relative energies.

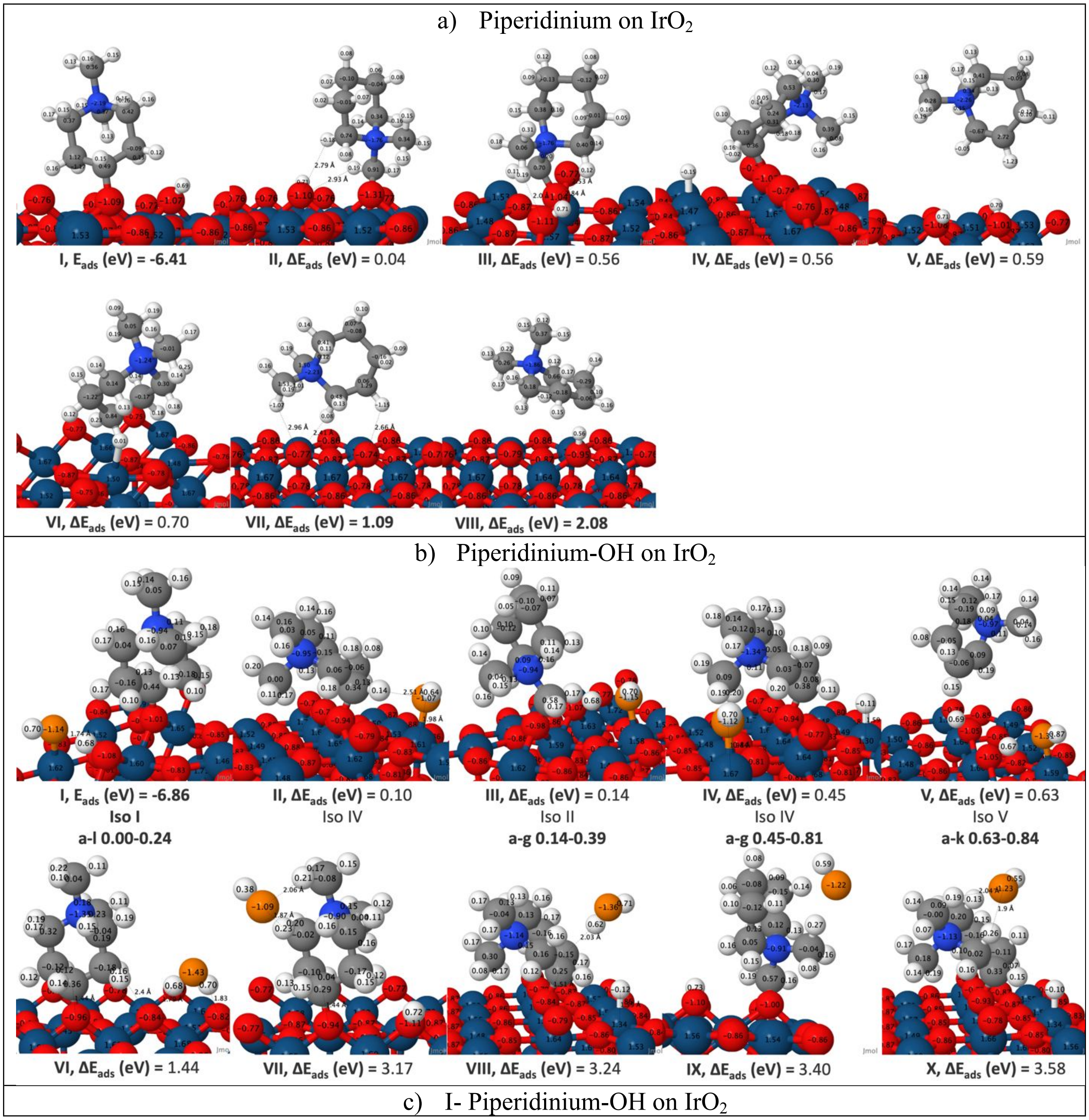

a) Piperidinium on IrO_2
I, E_{ads} (eV) = -6.41
II, ΔE_{ads} (eV) = 0.04
III, ΔE_{ads} (eV) = 0.56
IV, ΔE_{ads} (eV) = 0.56
V, ΔE_{ads} (eV) = 0.59
VI, ΔE_{ads} (eV) = 0.70
VII, ΔE_{ads} (eV) = 1.09
VIII, ΔE_{ads} (eV) = 2.08
b) Piperidinium-OH on IrO_2
I, E_{ads} (eV) = -6.86
Iso I
a-l 0.00-0.24
II, ΔE_{ads} (eV) = 0.10
Iso IV
III, ΔE_{ads} (eV) = 0.14
Iso II
a-g 0.14-0.39
IV, ΔE_{ads} (eV) = 0.45
Iso IV
a-g 0.45-0.81
V, ΔE_{ads} (eV) = 0.63
Iso V
a-k 0.63-0.84
VI, ΔE_{ads} (eV) = 1.44
VII, ΔE_{ads} (eV) = 3.17
VIII, ΔE_{ads} (eV) = 3.24
IX, ΔE_{ads} (eV) = 3.40
X, ΔE_{ads} (eV) = 3.58
c) I- Piperidinium-OH on IrO_2

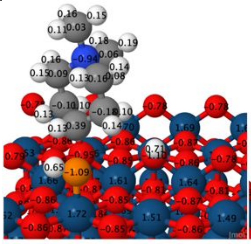

I-b, ΔE_{ads} (eV) = 0.17

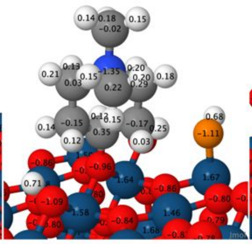

I-c, ΔE_{ads} (eV) = 0.18

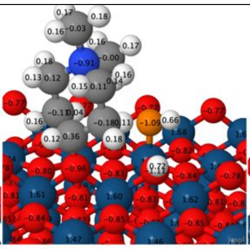

I-d, ΔE_{ads} (eV) = 0.19

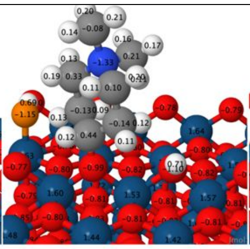

I-e, ΔE_{ads} (eV) = 0.20

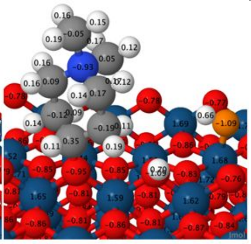

I-f, ΔE_{ads} (eV) = 0.20

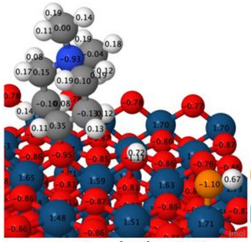

I-g, ΔE_{ads} (eV) = 0.20

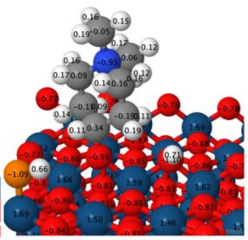

I-h, ΔE_{ads} (eV) = 0.21

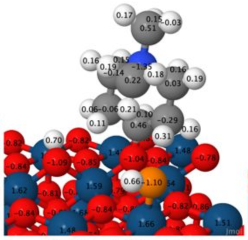

I-i, ΔE_{ads} (eV) = 0.22

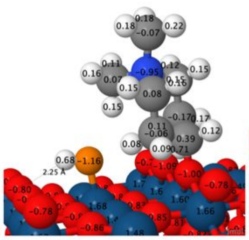

I-j, ΔE_{ads} (eV) = 0.22

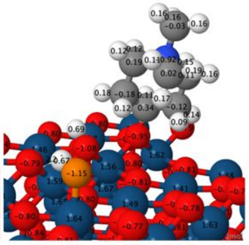

I-k, ΔE_{ads} (eV) = 0.23

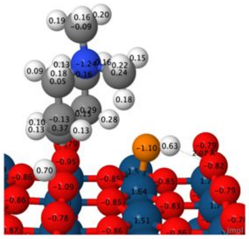

I-l, ΔE_{ads} (eV) = 0.24

d) III-Piperidinium-OH on IrO_2

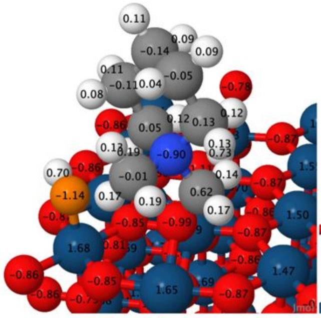

III-b, ΔE_{ads} (eV) = 0.30

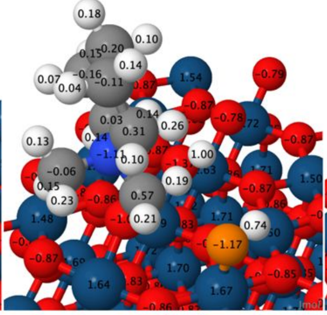

III-c, ΔE_{ads} (eV) = 0.32

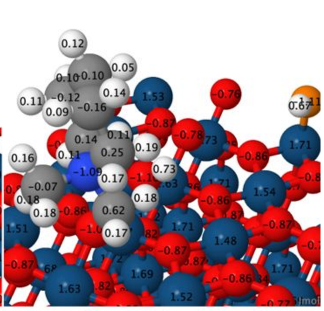

III-d, ΔE_{ads} (eV) = 0.32

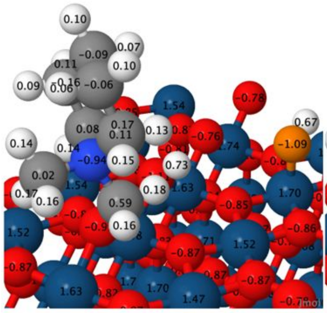

III-e, ΔE_{ads} (eV) = 0.34

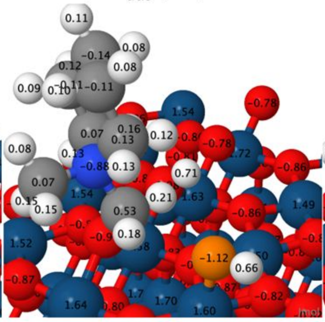

III-f, ΔE_{ads} (eV) = 0.34

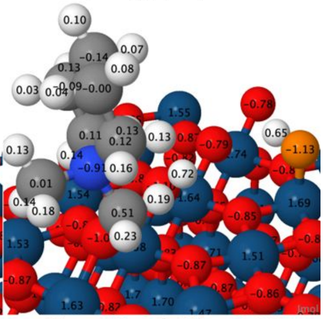

III-g, ΔE_{ads} (eV) = 0.39

e) IV-Piperidinium-OH on IrO_2

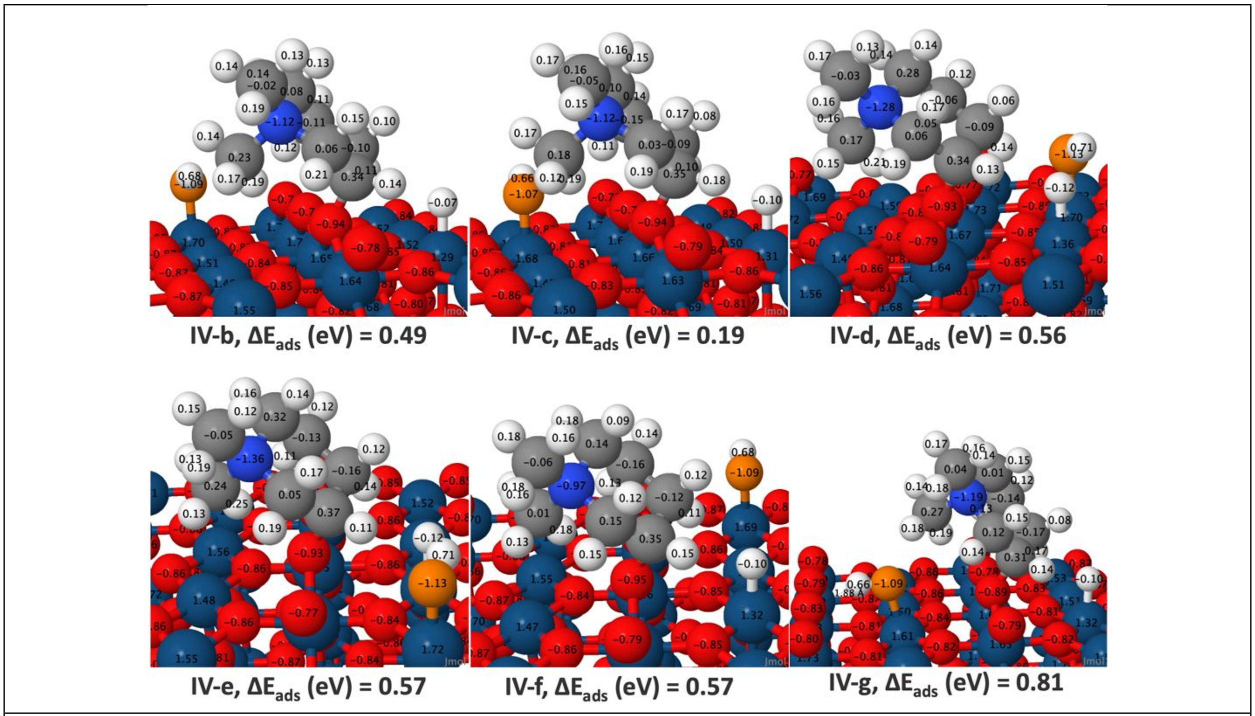

f) V-Piperidinium-OH on IrO_2

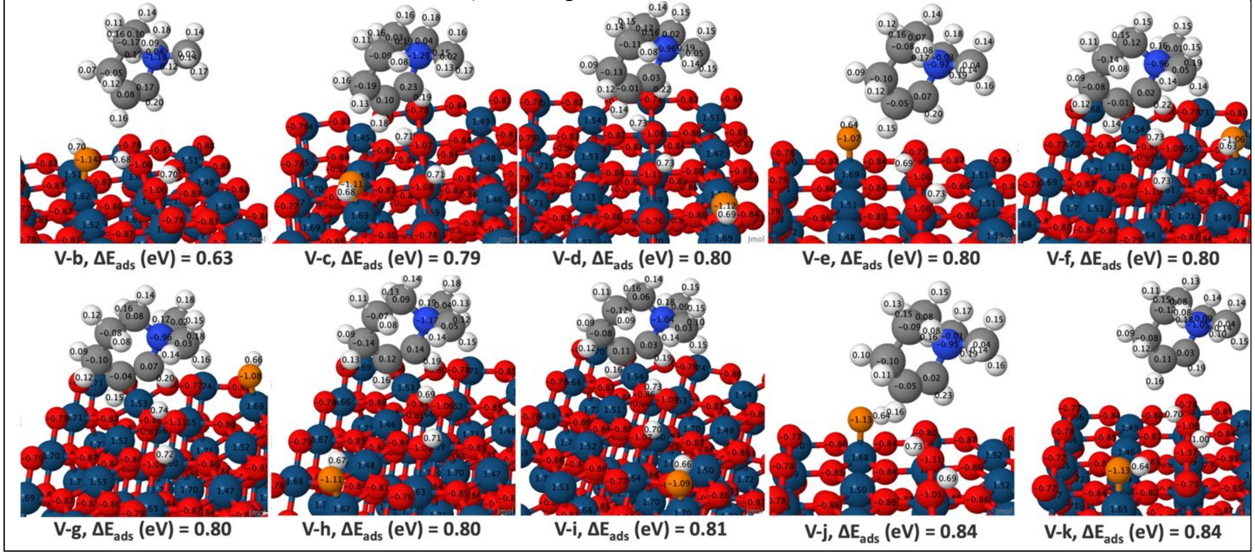

Figure S9. Piperidinium and Piperidinium-OH isomers on IrO_2 (110) with Bader charges and relative energies.

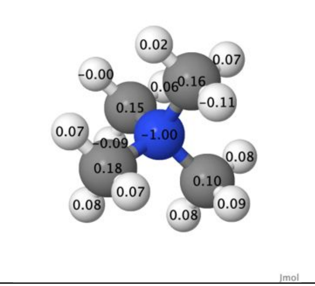	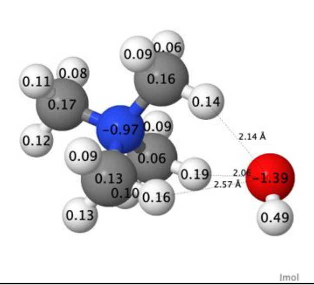
Iso I, $N(CH_3)_4$<br>E_{tot} (eV) = -85.800	Iso I, $N(CH_3)_4$-OH<br>E_{tot} (eV) = -98.003<br>**Δ**Q (e) = ±0.90

Figure S10. $N(CH_3)_4$ and $N(CH_3)_4$-OH isomers with Bader charges, bond distances, and total energies.

a) $N(CH_3)_4$ on NiO

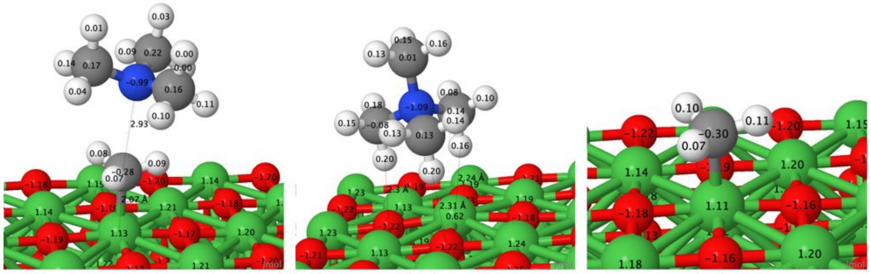

b) $N(CH_3)_4$-OH on NiO

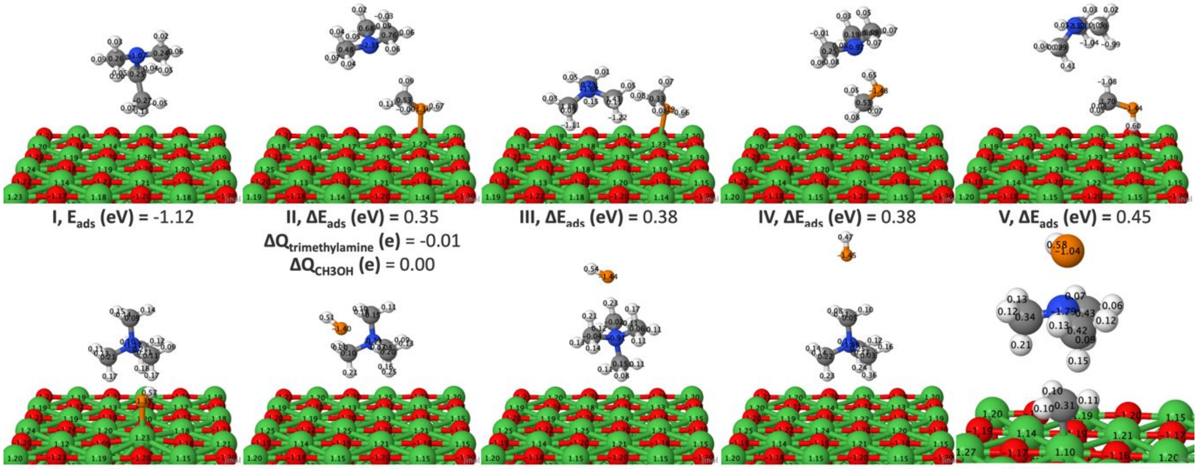

c) VI-$N(CH_3)_4$-OH on NiO

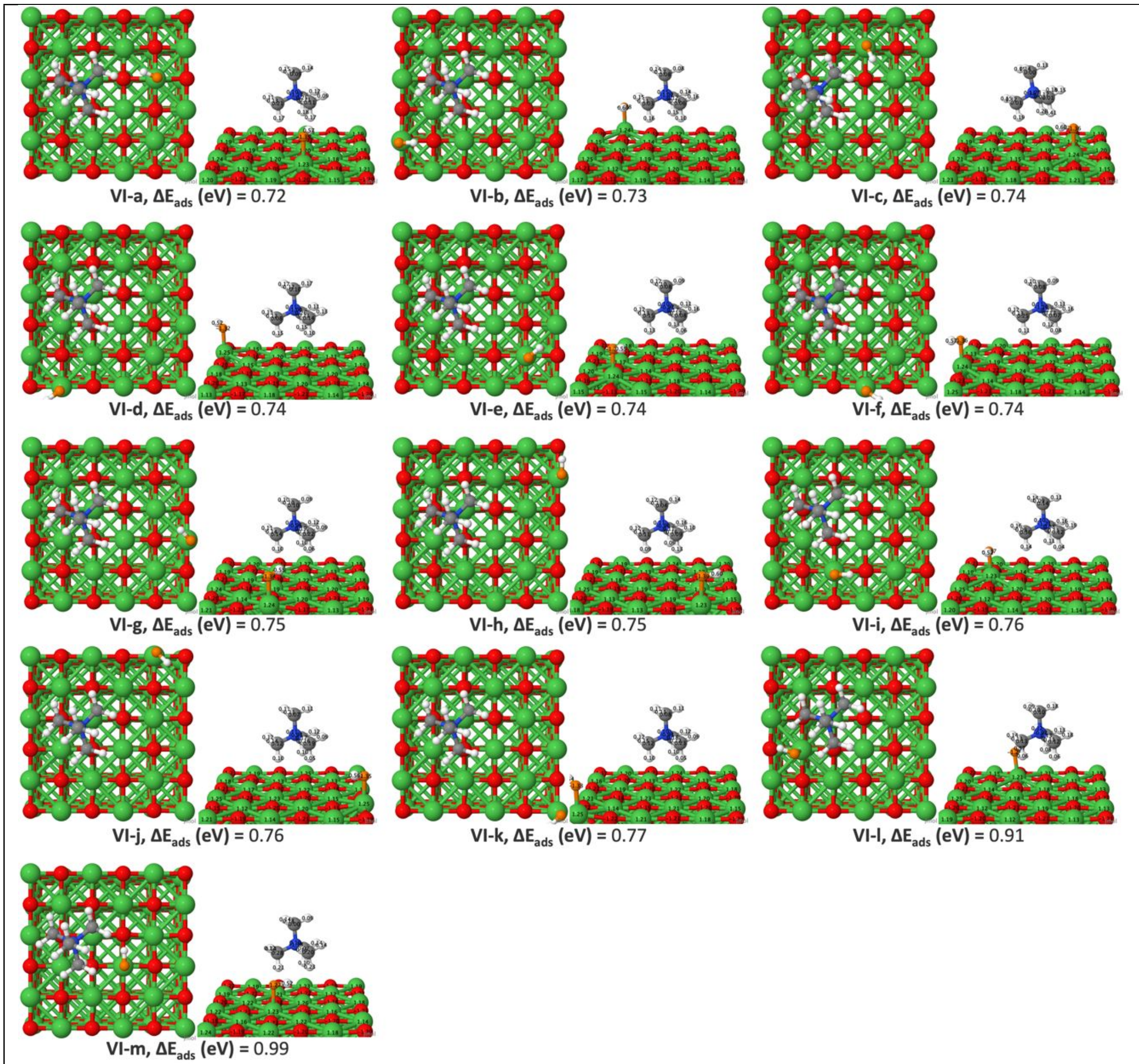

Figure S11. $N(CH_3)_4$ and $N(CH_3)_4$-OH isomers on NiO (100) with Bader charges and relative energies.

a) $N(CH_3)_4$ on IrO_2

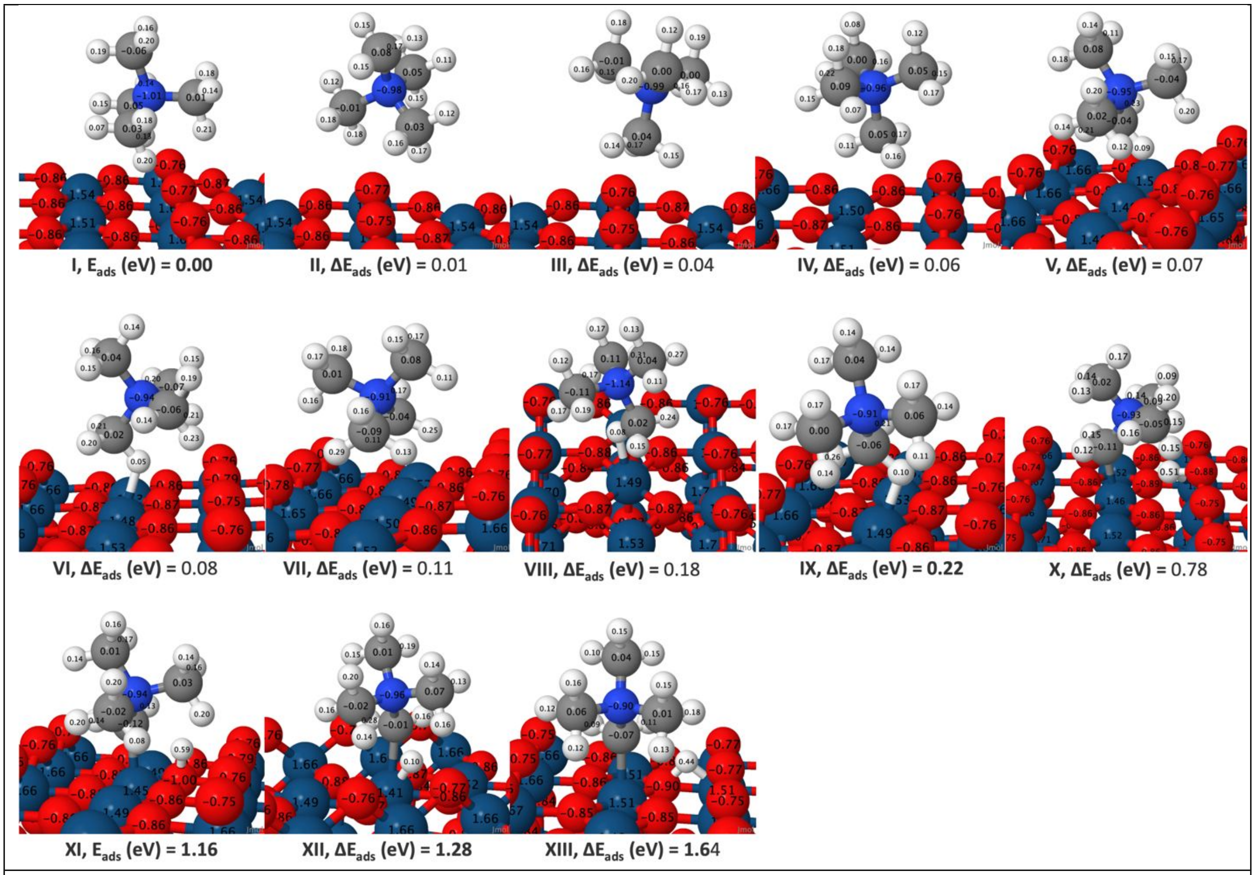

b) $N(CH_3)_4$-OH on IrO_2

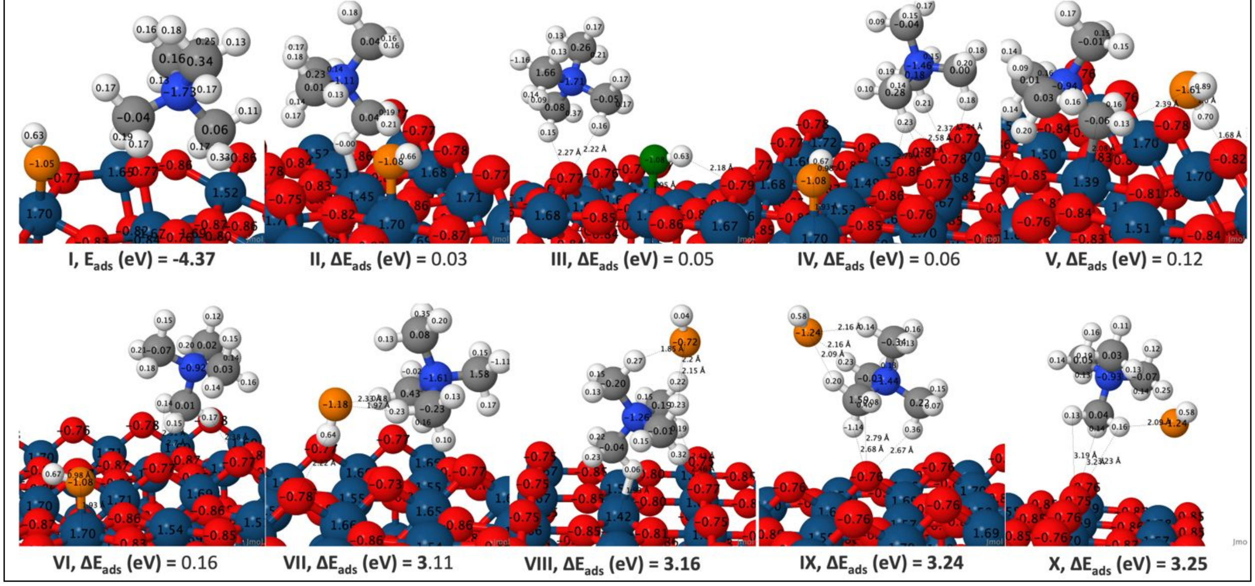

Figure S12. $N(CH_3)_4$ and $N(CH_3)_4$-OH isomers on IrO_2 (110) with Bader charges and relative energies.

Experimental

The thermodynamic potential for electrochemical water splitting was corrected for nonstandard temperature and pressure conditions as follows:[1, 2]

$$E_o = 1.229 - \frac{[T-298.15]\Delta S}{nF} + \frac{RT}{nF}\ln\left(\frac{P_{O_2}P_{H_2}^2}{P_o^3}\right) \text{ Eq. 1}$$

Where T is the testing temperature in K (in this work, 25 °C), $\Delta S = 2S_{H_2} + S_{O_2} - 2S_{H_2O}$, R is the gas constant (8.314 J/mol K), n is the number of moles of electrons involved in the electrochemical reaction (4 for OER), F is Faraday's constant (96485 C / mol e^-), the partial pressures of O_2 and H_2 (P_{O_2}, P_{H_2}) in Denver are 82.2 kPa, and the standard pressure (P_o) is 101.2 kPa. The thermodynamic potential is therefore **1.225 V** at the studied temperature and pressure conditions.

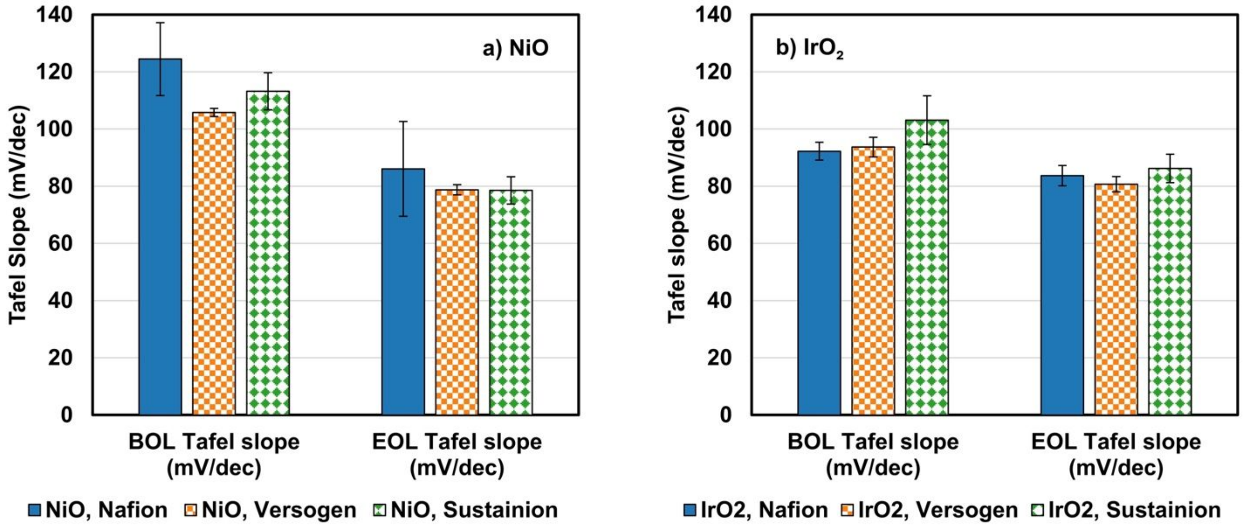

Figure S13. Tafel slopes before and after testing for a) NiO and b) IrO_2 with Nafion, Versogen, and Sustainion ionomers.

References

(1) Faulkner, L. R.; Bard, A. J. *Electrochemical methods: fundamentals and applications*; John Wiley and Sons, 2002.

(2) Fuller, T. F.; Harb, J. N. *Electrochemical engineering*; John Wiley & Sons, 2018.
